# Knowledge, Attitudes and Practices Regarding Prenatal Screening and Genetic Diagnosis of Chromosomal Anomalies Among Health Workers in Turkistan Region, Kazakhstan: A Cross-Sectional Survey of a Predominantly Primary-Care Workforce

**DOI:** 10.3390/healthcare14213776

**Published:** 2026-11-05

**Authors:** Zhansaya Torgauytova, Ardak Ayazbekov, Sundetgali Kalmakhanov, Almagul Kurmanova, Seçil Özkan, Saken Khaidarov, Gaukhar Kurmanova, Arman Nurmakhanbetov, Aknur Turgumbayeva, Amina Abdrakhmanova, Diana Sundetova, Shynar Zakaryanova, Elnur Adilova

**Affiliations:** 1 Department of Public Health, Khoja Akhmet Yassawi International Kazakh-Turkish University, Turkistan 161200, Kazakhstan; jans.86@mail.ru; 2 Scientific Center of Obstetrics, Gynecology and Perinatology, 125 Dostyk Ave., Almaty 050010, Kazakhstan; ardakayazbekov966@gmail.com; 3 Faculty of Medicine and Healthcare, Farabi University, 71, Al-Farabi Av., Almaty 050040, Kazakhstan; kurmanova.almagul@kaznu.kz (A.K.); kurmanova.gaukhar@kaznu.kz (G.K.); aknur.turgumbayeva@kaznu.edu.kz (A.T.); amisaparova@gmail.com (A.A.); diana.sundetova@kaznu.edu.kz (D.S.); 4 Department of Public Health, Faculty of Medicine, Gazi University, Ankara 06500, Türkiye; ozkans@gazi.edu.tr; 5 Faculty of Philosophy and Political Science, Farabi University, 71, Al-Farabi Av., Almaty 050040, Kazakhstan; arman.nurmakhanbetov@kaznu.edu.kz (A.N.); zakaryanova.shynar@kaznu.kz (S.Z.); adilova.elnur@kaznu.kz (E.A.)

**Keywords:** prenatal screening, prenatal diagnosis, chromosome aberrations, trisomy, health knowledge, attitudes, practice, primary health care, nurses, midwifery, genetic counselling, Kazakhstan

## Abstract

Background/Objectives: In Kazakhstan, pregnant women reach prenatal screening mainly through primary care, where nurses and midwives make many of the routine antenatal contacts. How well this workforce knows the screening pathway has not been measured. We assessed knowledge, attitudes and practices (KAP) regarding prenatal screening and genetic diagnosis among health workers in antenatal care. Methods: A cross-sectional online survey of 1331 health workers in the Turkistan region (June–July 2026) was conducted. The questionnaire was developed for this study, mapped to the national orders, and has not been externally validated. Knowledge was summarised as an index of 12 factual items and self-reported practice as an index of seven items; attendance at genetics training was analysed as an exposure, and attitude items were reported individually. We used Wilson intervals, nonparametric tests and logistic regression with robust standard errors. Results: Nurses made up 67.8% of respondents and 86.1% worked in primary care. Respondents answered 54.1% of the knowledge items correctly on average (55.2% after removing one item with an incorrect answer key), and 14.6% reached the 80% threshold. Only 16.4% knew that the combined-screening results are interpreted as multiples of the median. Over 84% supported screening and referral, but 38.8% felt fully confident in their own knowledge. Good practice was reported by 42.3%. Recent genetics training was associated with good practice (adjusted OR 2.54, 95% CI 1.90–3.40) but not with good knowledge (0.89, 0.63–1.26). Conclusions: Support for prenatal genetic services is broad, while factual knowledge is limited and concentrated in a few items. Training attendance was linked to reported practice but not to knowledge, and the design cannot show why. Training for primary-care staff should be evaluated by knowledge testing rather than attendance.

## 1. Introduction

Congenital disorders remain one of the least tractable causes of perinatal and child death. The World Health Organisation estimates that about 240,000 newborns die within 28 days of birth each year from congenital conditions, with a further 170,000 deaths between one month and five years of age, and that nine in ten children with a severe congenital disorder are born in low- and middle-income countries [1]. In 2010 the World Health Assembly asked member states to build national capacity for the prevention and care of birth defects, including the training of health workers [2]. Because deaths from infection and intrapartum complications have fallen faster, congenital disorders now account for a growing share of neonatal and under-five deaths [3,4]. Chromosomal anomalies, trisomy 21 above all, are the group that prenatal screening targets most directly, and they are the focus of this survey.

International guidance recommends that every pregnant woman, whatever her age, be offered screening for the common trisomies after counselling, and that the decision to accept it be hers [5]. The usual first-trimester approach is the combined test: an ultrasound examination at 11^+0^–13^+6^ weeks that includes nuchal translucency, together with maternal serum pregnancy-associated plasma protein A (PAPP-A) and free β-human chorionic gonadotropin (β-hCG). The biochemical values are expressed as multiples of the median (MoM) and combined with maternal age and the ultrasound findings into an individual risk [6,7,8]. Cell-free DNA analysis of maternal blood detects trisomy 21 with much higher sensitivity and is used either as a first-line or as a contingent test [5,9,10]. A mid-trimester anomaly scan follows at 18–22 weeks [11], and chorionic villus sampling or amniocentesis is offered when the risk is high. Each step depends on the one before it. A scan done outside its window cannot be combined with the biochemistry, a MoM value the clinician cannot read cannot be explained to the woman, and a high risk that never reaches a geneticist changes nothing.

In Kazakhstan, the pathway is set out in three Ministry of Health orders, all in force at the time of the survey. Order No. 704 of 9 September 2010 on the rules for organising screening, in its current version, describes the three-stage prenatal programme and the combined first-trimester risk calculation, and lists the indications for referral to a medical geneticist: a combined risk of 1:150 or higher, maternal age of 37 years or more, or a previous pregnancy with a chromosomal or genetic disorder [12]. Order No. ҚR DSM-54 of 21 June 2022 (amended 21 February 2025) fixes the ultrasound windows at 11^+0^–13^+6^, 19^+0^–21^+0^ and 30^+0^–32^+6^ weeks [13], and Order No. ҚR DSM-92 of 26 August 2021 (amended 2022 and 2025) sets the standard of obstetric and gynaecological care [14]. Neonatal screening has run nationally since 2007 [15]. Specialist genetic services are concentrated in Almaty and Astana. Turkistan city lies about 700 km in a straight line from Almaty and about 900 km from Astana, and road distances are longer (Supplementary Figure S1). In 2024 Turkistan region had the highest crude birth rate in the country, 24.9 per 1000 population [16], so screening volumes are large and referral distances are long. The region is also culturally mixed, with a large Uzbek community alongside the Kazakh majority, and decisions about screening, invasive testing and termination are often taken with the extended family and shaped by religious views.

**Figure 1:**
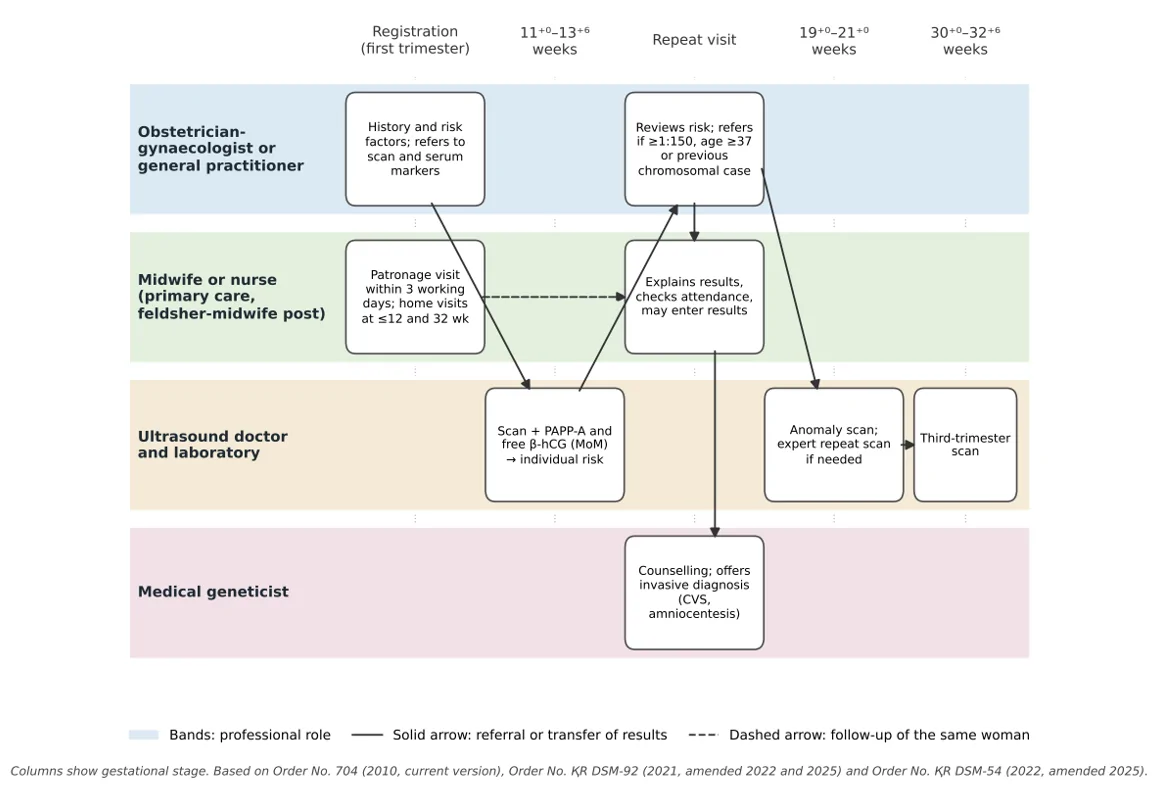
Organisation of prenatal screening for chromosomal anomalies in Kazakhstan by professional role and gestational stage, as set out in the national orders [12,13,14]. PAPP-A, pregnancy-associated plasma protein A; β-hCG, free β-human chorionic gonadotropin; MoM, multiple of the median; CVS, chorionic villus sampling. Coloured bands show professional roles and columns show gestational stages; solid arrows show referral or transfer of results, and the dashed arrow shows follow-up of the same woman.

Who does what along this pathway matters for how a survey like ours is read (Figure 1). The first antenatal visit is with an obstetrician-gynaecologist or a general practitioner, who registers the pregnancy in the first trimester, takes the history and refers the woman for the first-trimester scan and serum markers; at the repeat visit, the same physicians refer women who meet the criteria to a medical geneticist [12,14]. Scans are performed by certified ultrasound doctors [13]. Midwives and nurses follow physiological pregnancies and make the patronage home visits, the first within three working days of registration and then at up to 12 and at 32 weeks, and midwives may enter screening results into the information system [12,14]. Nurses and midwives therefore meet most women early, usually within the first trimester, while the formal decisions to refer to a geneticist or to request invasive testing rest with physicians. Their role is to explain the screening, make sure appointments happen and pass results on, which still requires knowing the windows, the tests and what a result means. In rural feldsher-midwife posts a midwife or nurse is often the only health worker on site and arranges referral to the district physician.

Knowledge, attitudes and practices (KAP) surveys on prenatal screening have mostly been carried out among pregnant women [17,18]. Provider surveys exist but have concentrated on obstetricians and on the introduction of non-invasive prenatal testing (NIPT) in high-income systems [19,20,21], with few from lower-middle-income settings [22]. Studies of nurses and midwives point to low genomic literacy across countries [23,24,25]. We found no provider data from Central Asia in PubMed, Scopus, eLibrary.ru, CyberLeninka and DergiPark, searched in English, Russian, Kazakh and Turkish, and no study that includes the nurses and midwives who make most first contacts in antenatal care.

We therefore surveyed health workers involved in antenatal care in Turkistan region. We aimed to measure objective knowledge of prenatal screening and genetic diagnosis at the item level, to describe attitudes, perceived accessibility and barriers, to quantify self-reported practice, and to identify factors associated with good knowledge and good practice. Because no validated Kazakhstan-specific instrument existed, we also report how our questionnaire behaved psychometrically, so that it can be criticised and improved rather than reused as it stands.

## 2. Materials and Methods

### 2.1. Study Design and Setting

This cross-sectional online survey was conducted from 1 June to 30 July 2026. Invitations with a link to the form were distributed through the regional health administration to clinical staff at facilities of all levels in the Turkistan region, southern Kazakhstan: the regional perinatal centre and level 1 to 3 perinatal units, district and city polyclinics, primary health care centres, district hospitals and rural feldsher-midwife posts. Reporting follows the STROBE recommendations for cross-sectional studies [26] and the CHERRIES checklist for internet surveys [27]; the completed CHERRIES checklist is given in Supplementary Table S2.

### 2.2. Participants

Eligible respondents were clinical staff with any role in antenatal care: nurses, midwives, general practitioners, obstetrician-gynaecologists, neonatologists, geneticists and ultrasound specialists. The questionnaire was self-administered through Google Forms (Google LLC, Mountain View, CA, USA); participation was voluntary and no incentive was offered. The form did not record how many people opened the link, so view and participation rates cannot be calculated. All items were mandatory, so a form could be submitted only when complete; 1331 forms were submitted and none was excluded. Because recruitment was open rather than drawn from a register, a response rate cannot be calculated, and we did not obtain a regional workforce denominator, so coverage cannot be estimated either. We treat the sample as a convenience sample of the regional workforce.

The form was offered in Kazakh only. The regional health administration distributes staff surveys in the state language, and no validated Russian version existed at the time. Most health workers in the region trained in Kazakh, but some work mainly in Russian or Uzbek; they may have been less likely to take part or may have read some items differently. We return to this among the limitations.

### 2.3. Questionnaire Development

The study team wrote the instrument because no validated Kazakhstan-specific tool was available. The form has 38 items: 35 content items in four survey blocks and three items on consent, profession and workplace. By domain, the content items fall into three groups. Knowledge has 14 items: 11 single-choice factual items and three multiple-response questions (regulatory documents, diagnostic methods and the components of combined screening). Attitudes and perceptions have 11: five on support for screening services, one on self-rated confidence, and perceived accessibility, perceived public awareness, awareness of a local genetics service, barriers and the termination item. Practice has 10: eight frequency items, the main source of information and the reasons for not referring for invasive diagnosis. Barriers and reasons for not referring also allowed several answers. Item content was mapped onto the national orders so that correct answers reflect Kazakh practice, and two obstetrician-gynaecologists and one public health specialist reviewed the items for clarity before distribution. The instrument was written in Kazakh; for this paper it was translated into English by Z.T. and back-translated by an independent bilingual public health specialist, with discrepancies resolved by consensus. The full instrument in Kazakh and English is given in Supplementary Material S1.

Four item formats need stating explicitly. The first-trimester timing item asked in which week of pregnancy the first prenatal screening ultrasound is performed, so it referred to the scan and not to blood sampling; the options were 9^+0^–12^+6^, 10^+0^–14^+6^, 11^+0^–13^+6^ and 12^+0^–14^+6^ weeks. The item on the components of combined first-trimester screening allowed several answers from six options: ultrasound, PAPP-A and β-hCG, amniocentesis, unconjugated estriol, alpha-fetoprotein and inhibin A, and visual follow-up of the pregnancy. Maternal age, which enters the risk algorithm, was not offered as a component. The practice items had no “not applicable to my role” option, so a respondent who did not perform an action answered “never” and scored zero. The training question asked “Have you attended training or seminars on medical genetics in the last 3 years?” with the answers yes or no; it recorded no content, duration or provider.

### 2.4. Scoring Framework

Table 1 lists every item that enters the two indices and how it was scored. The knowledge index has 12 factual items, one point each (range 0–12): ten single-choice items, the multiple-response item on combined screening, scored as correct only when ultrasound and the PAPP-A/β-hCG option were selected and nothing else, and the maternal-age item. The ultrasound windows were keyed to Order No. ҚR DSM-54 [13]. The maternal-age item needs a separate word. Its key in the analysis was 35 years, but Orders No. 704 and No. ҚR DSM-92 set the referral age at 37 years [12,14], and in a combined-screening pathway age is one input into an individual risk rather than an indication on its own [5]. The error lay in the answer key used for the analysis, not in data collection: the item and its five options (30, 32, 35, 37 and 40 years) were presented as designed, and the recorded answers are unaffected. The respondent-level knowledge index, its categories and the knowledge model were computed with this key and were not refitted without the item. We therefore report the item’s answers separately and give the mean of the remaining 11 items, which can be computed exactly from the item counts. The same applies to a second-trimester version in which 18^+0^–21^+6^ weeks, close to international guidance for the anomaly scan [11], is also accepted. The two other multiple-response knowledge questions (regulatory documents and diagnostic methods) are reported descriptively.

The practice index has seven frequency items coded 0, 1 or 2 (range 0–14; Table 1). Attendance at genetics training is an exposure, not a practice, so it is not part of the index and is used only as a predictor; an eight-item version that counts attendance as 2 points is reported as a sensitivity analysis. Attitude items are reported one by one and are not summed, for three reasons. The response options ranked “mandatory” above “recommended” for screening, invasive diagnosis and referral, so a respondent whose answer matched informed choice would have scored lower than one whose answer did not. One item measured confidence in one’s own knowledge, which is a different construct. And a summed score had a marked ceiling, with a mean of 83% of the maximum (Supplementary Table S5).

For both indices, scores below 60% of the maximum were classified as poor, 60–79.9% as moderate and 80% or above as good. These cut-points make the domains easier to compare, but they are operational definitions and not validated competency standards. Free-text answers that matched no predefined category were coded conservatively, and uninterpretable answers scored zero.

### 2.5. Statistical Analysis

Categorical variables are reported as counts and percentages with Wilson 95% confidence intervals [28], and indices as means with standard deviations and medians with interquartile ranges. Internal consistency is reported as Cronbach’s alpha, which for binary items equals KR-20 [29]. Factorability was assessed with the Kaiser–Meyer–Olkin (KMO) measure, Bartlett’s test of sphericity and the eigenvalues of the Pearson item correlation matrix. Groups were compared with the Kruskal–Wallis test and epsilon-squared (three or more groups) or the Mann–Whitney U test and the rank-biserial correlation (two groups). Associations between indices were assessed with Spearman’s rho.

Logistic regression estimated adjusted odds ratios (aOR) for good knowledge and good practice in all 1331 respondents. Both models included professional group (nurses as reference), workplace (primary care as reference), genetics training in the previous three years, clinical guidelines or conferences as the main information source, awareness of a local genetics service (three levels, entered per level) and perceived accessibility (1–5, per point). The practice models also included the knowledge index and the summed attitude score. Both may lie on the path from training to practice (Supplementary Figure S2), so adjusting for them is more likely to shrink the training estimate than to inflate it. Standard errors are heteroskedasticity-consistent sandwich (HC1) estimates. No facility or district identifiers were collected, so clustering could not be modelled and the standard errors are probably too small. Fit is reported as McFadden’s pseudo-R^2^.

Three sensitivity analyses were performed. The first repeated the main estimates after keeping a single record for each of the 13 repeated response profiles. The second refitted the practice model with the eight-item index, in which training attendance counts as a practice, to show how much of the original association was built into the score. The third recomputed the mean knowledge index without the maternal-age item and with both second-trimester answers accepted; these means are exact sums of the item proportions.

Analyses were run in Python 3.12.3 (Python Software Foundation, Wilmington, DE, USA) with the open-source libraries pandas 3.0.2, NumPy 2.4.4, SciPy 1.17.1 and statsmodels 0.14.6, and figures were drawn with Matplotlib 3.10.8. All tests were two-sided with *p* < 0.05 as the significance level, and effect sizes and confidence intervals were given more weight than *p*-values.

### 2.6. Ethics

The study was approved by the Local Bioethics Commission of Khoja Akhmet Yassawi International Kazakh-Turkish University (decision of 8 May 2026, protocol No. 4/26; written conclusion No. 216 of 29 May 2026, valid until 8 May 2027). The commission reviewed the survey as part of the doctoral project of Z.T. on medical genetic care for pregnant women in Turkistan region, whose topic was approved by university order No. TO 223 of 25 December 2025. The commission is certified by the Forum of Ethics Committees of Kazakhstan (KazREC) for compliance with national GCP standards, WHO guidance on ethics review and ICH GCP (registration No. XXV-R, valid from 5 December 2025 to 5 December 2028). The conclusion with an English translation and the certificate are provided as Supplementary Material S2. The opening page of the form described the purpose of the survey and the intended use of the data, and respondents gave electronic consent before proceeding. No names, contact details, facility identifiers or other potentially identifying information was collected.

The sequence of events was: KazREC certification of the commission, 5 December 2025; approval of the dissertation topic, 25 December 2025; ethics decision, 8 May 2026, and written conclusion, 29 May 2026; data collection, 1 June to 30 July 2026, entirely after approval; analysis, after the close of data collection. The answer key and the description of the national pathway follow the versions of the orders in force during data collection: Order No. 704 as amended on 21 August 2025, Order No. ҚR DSM-92 as amended on 7 December 2022 and 21 February 2025, and Order No. ҚR DSM-54 as amended on 21 February 2025 [12,13,14].

## 3. Results

### 3.1. Respondents and Data Quality

None of the 1331 submitted forms had missing item-level responses. The export contained one additional column, labelled as an email address, that held the value 39 in every record. It was generated by the form platform’s email-collection setting, which was switched off; the value is a system code and not respondent data. It was excluded. Thirteen response profiles occurred more than once, accounting for 19 records beyond the first occurrence of each profile. All submissions had distinct timestamps and no respondent identifier was collected, so these records could not be told apart from genuinely identical answer patterns; they were kept in the primary analysis and removed in a sensitivity analysis.

Nurses made up two thirds of respondents and obstetrician-gynaecologists fewer than one in twenty, and 86.1% worked in primary care (Table 2). One respondent in eight did not know whether a medical genetics service operated in their area, and one in three had attended genetics training in the previous three years.

### 3.2. Knowledge of Prenatal Screening and Genetic Diagnosis

**Figure 2:**
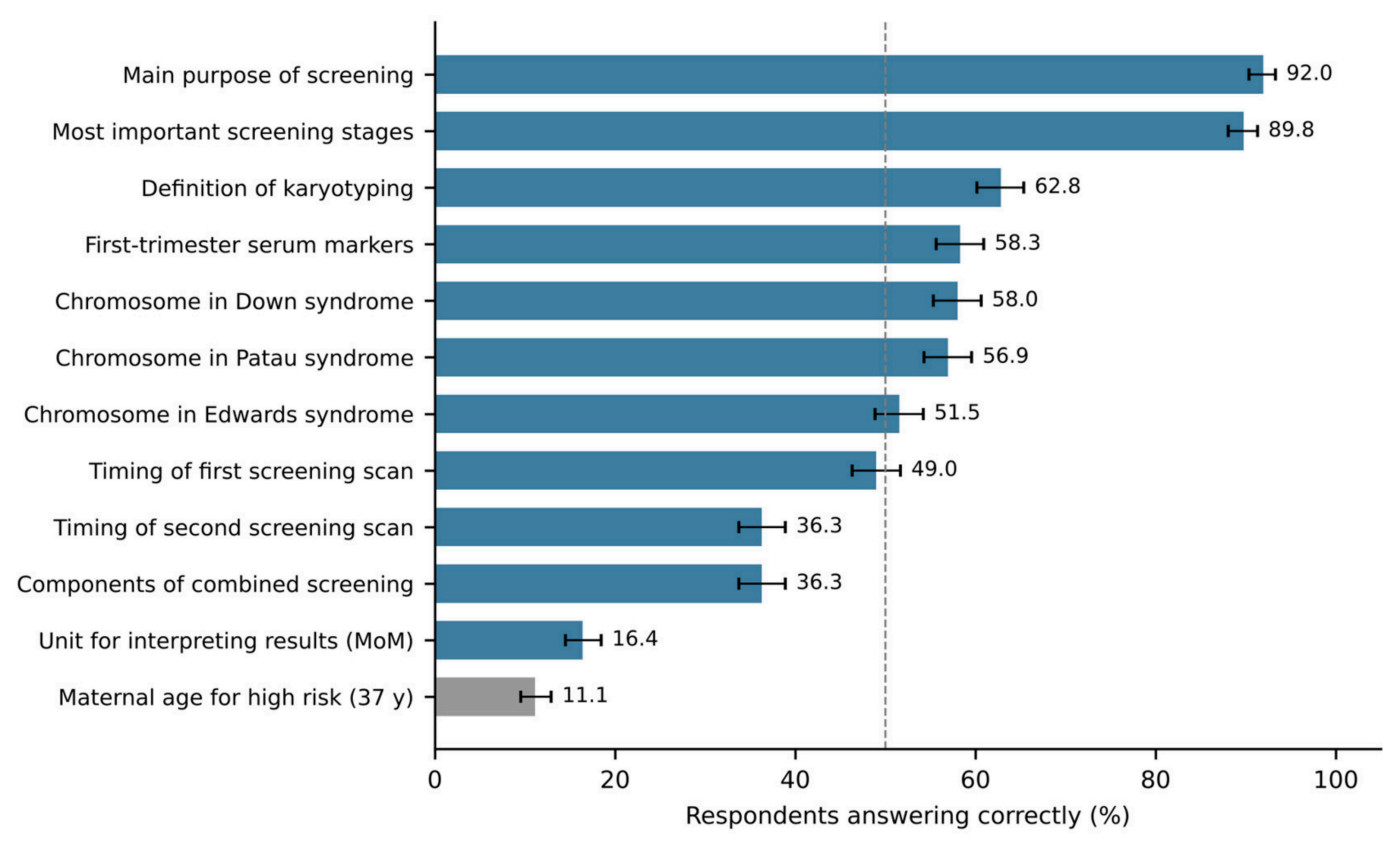
Proportion of respondents answering each knowledge item correctly, with Wilson 95% confidence intervals (*n* = 1331). The maternal-age item (grey) is shown against the national key of 37 years. The dashed line marks 50%.

Respondents answered on average 6.49 ± 2.62 of the 12 knowledge items correctly (54.1% of the maximum; median 7, interquartile range 4–9). Without the maternal-age item, the mean of the remaining 11 items is 6.07 (55.2%). Item-level results are shown in Table 3 and Figure 2.

Almost everyone knew the purpose of screening and the trimesters in which it matters, but performance dropped on the technical items. Fewer than half gave the national first-trimester window; the most common wrong answer, 9^+0^–12^+6^ weeks, may reflect the earlier start of blood sampling. About a third gave the national second-trimester window, and 401 respondents (30.1%) chose 18^+0^–21^+6^ weeks; with both answers accepted, 884 (66.4%, 95% CI 63.8–68.9) answered correctly. The weakest item concerned how combined-screening results are interpreted: 16.4% chose MoM and more than twice as many chose IU/L. On the maternal-age item, 41.3% chose 35 years and 31.2% chose 40 years; only 148 (11.1%) chose 37 years, the age set in the national orders, and 13.6% chose 30 years. Item results by level of care are given in Supplementary Table S3.

Awareness of diagnostic methods was uneven: respondents recognised on average 2.0 of five listed methods. Ultrasound was named by 77.2% and biochemical screening by 61.2%, but molecular genetic testing by 27.3%, invasive diagnosis by 22.2% and NIPT by 11.0%. Order No. ҚR DSM-92 was recognised by 70.2% of respondents, Order No. 704 by 30.5% and Order No. ҚR DSM-54 by 29.8%, while 12.7% and 7.4% selected the two distractor documents; only 97 respondents (7.3%) chose all three relevant orders and no distractor (Supplementary Table S1).

### 3.3. Attitudes, Perceived Accessibility and Barriers

Support for the services was close to universal (Table 4). More than 84% regarded screening for all pregnant women and referral of high-risk pregnancies to a geneticist as obligatory, almost nine in ten saw genetic counselling as beneficial, and 92.5% considered invasive diagnosis necessary when indicated. Confidence was lower: 38.8% described themselves as fully confident in their knowledge of prenatal genetic screening, and 57.7% said they knew the main principles but needed further training. The counselling question offered only “yes” and “no”; the few intermediate answers are free-text replies such as “maybe”, so the item is in effect binary.

**Figure 3:**
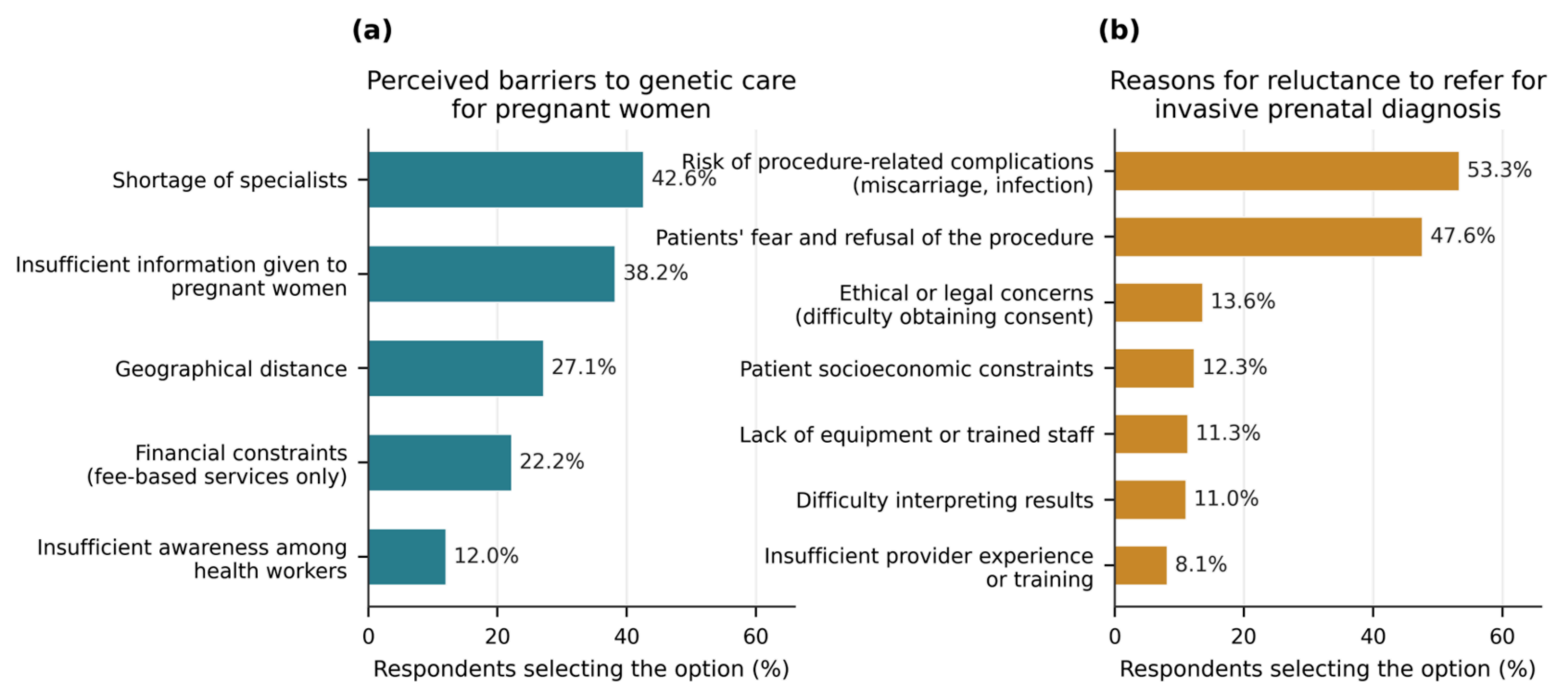
Perceived barriers to genetic care for pregnant women (**a**) and reasons for reluctance to refer for invasive prenatal diagnosis (**b**). Both questions allowed multiple answers, so percentages sum to more than 100.

Accessibility of medical genetic care in the respondent’s own region was rated 4 or 5 out of 5 by 64.9%. Two questions dealt with information, and they asked about different people. Asked to rate the general population’s awareness of what genetic services offer, 44.9% answered high, 47.7% moderate and 7.4% low. Asked separately about barriers for pregnant women, 38.2% named inadequate information given to pregnant women. The barriers named most often were a shortage of specialists (42.6%), inadequate information for pregnant women (38.2%), geographical distance (27.1%) and financial constraints (22.2%) (Figure 3a).

Medically indicated termination of pregnancy for a severe untreatable inherited condition was considered appropriate by 77.3% of respondents; 19.1% could not answer and 3.6% disagreed.

### 3.4. Clinical and Counselling Practice

The mean practice index was 10.12 ± 3.33 of 14 (72.3% of the maximum). Recommending molecular genetic testing and preconception counselling were the actions reported most consistently, and referral to a medical geneticist the least (Table 5). Attendance at genetics training, analysed as an exposure, was reported by 439 respondents (33.0%).

Half of the respondents named clinical guidelines as their main source of information about prenatal screening; the rest relied on colleagues, the internet or conferences (Table 2).

The most common reasons for not referring a woman for invasive diagnosis were the risk of procedure-related complications (53.3%) and patients’ fear or refusal (47.6%). Ethical or legal difficulties, cost, lack of equipment or staff, difficulty interpreting results and the respondent’s own limited experience were each named by 8–14% (Figure 3b).

One inconsistency stands out. NIPT was named as a known method by only 147 respondents (11.0%), yet 844 (63.4%) said they always recommend it when indicated, including 733 of the 1184 (61.9%) who had not listed it.

Referral to a geneticist, receiving and applying genetic results and recommending NIPT or molecular testing are formally physician tasks (Figure 1). Practice item responses are available by level of care but not by profession. Primary-care staff more often reported frequent referral to a geneticist (44% versus 34% of hospital and perinatal staff), regular use of genetic test results (50% versus 39%) and regular preconception counselling (72% versus 59%; Fisher’s exact test, *p* ≤ 0.004 for each; Supplementary Table S4).

### 3.5. Indices, Categories and Psychometric Properties

Good knowledge was reached by 194 respondents (14.6%, 95% CI 12.8–16.6) and 832 (62.5%) fell in the poor category. Good practice was reported by 563 (42.3%, 95% CI 39.7–45.0).

Cronbach’s alpha was 0.697 for the 12-item knowledge index and 0.826 for the seven-item practice index. The knowledge items were factorable (KMO 0.793; Bartlett χ^2^ = 2036.6, df 66, *p* < 0.001) but produced four eigenvalues above 1, which is what one expects of separate facts on timing, biochemistry and cytogenetics rather than one latent trait. The practice items were unidimensional (eight-item version: KMO 0.870; Bartlett χ^2^ = 2895.6, df 28, *p* < 0.001; one eigenvalue above 1).

Knowledge correlated only weakly with practice (Spearman’s rho 0.153; knowledge index and eight-item practice index).

### 3.6. Differences by Professional Group, Workplace and Training

Obstetrician-gynaecologists scored highest on knowledge and nurses lowest; differences by workplace were smaller (Table 6).

Respondents who had attended genetics training in the previous three years scored no better on knowledge than those who had not (6.44 ± 2.71 versus 6.51 ± 2.57; *p* = 0.522), but they reported more practice on the seven-item index (11.62 ± 2.48 versus 9.39 ± 3.46; Mann–Whitney *p* < 0.001, rank-biserial r = 0.39).

### 3.7. Factors Associated with Good Knowledge and Good Practice

Good knowledge was associated with being an obstetrician-gynaecologist rather than a nurse (aOR 5.46, 95% CI 2.93–10.19), being a midwife (1.66, 1.08–2.56), awareness of local genetic services (1.54 per level, 1.16–2.05) and perceived accessibility (1.24 per point, 1.07–1.44), and lower odds among hospital staff (0.38, 0.16–0.88). Recent training showed no association (0.89, 0.63–1.26), and the model explained little of the variation (pseudo-R^2^ 0.082) (Table 7).

On the seven-item practice index, recent training was associated with good practice (aOR 2.54, 1.90–3.40), as were being an obstetrician-gynaecologist (5.81, 2.86–11.82), relying on guidelines or conferences (2.20, 1.66–2.93) and a higher attitude score (1.62 per point, 1.47–1.78). On the eight-item index, which counts training attendance itself, the training estimate rose to 9.86 (7.10–13.69); the remaining terms of that model are given in full in Table 7, and knowledge was not independently associated with good practice (1.02, 0.97–1.08).

Perinatal-unit staff had the highest unadjusted practice scores (Table 6), yet after adjustment their odds of good practice were lower than those in primary care (aOR 0.23, 0.12–0.44, on seven items). Hospital and perinatal staff were more often trained (41% versus 32% in primary care), and the model holds training and profession constant. Once it does, several practice items describe routine primary-care work that hospital-based staff do less often. Item by item, primary-care staff more often reported frequent referral to a geneticist, regular use of genetic test results and regular preconception counselling (Supplementary Table S4).

### 3.8. Sensitivity Analyses

Removing the 19 duplicate records changed the mean knowledge and practice indices by less than 0.2 percentage points (Table 8).

Without the maternal-age item, the mean knowledge index was 55.2% of the maximum, and with both second-trimester answers also accepted it was 57.9%, against 54.1% for the index as analysed. Counting training attendance as a practice (eight items) raised the training estimate from 2.54 to 9.86, which shows how much of the original association was built into the score.

## 4. Discussion

Three findings stand out. Support for prenatal screening and for genetic services was close to unanimous. Objective knowledge was limited and uneven, with one respondent in seven reaching the operational good-knowledge threshold. And recent training, one of the strongest correlates of reported good practice, showed no association with what respondents knew.

### 4.1. High Endorsement Alongside Low Factual Accuracy

This pattern of strong endorsement and limited knowledge has been seen elsewhere. In England, nurses and midwives rated genomics as important but had little confidence in using it, and the gap widened over four survey years [24]. Australian nurses and midwives showed low genomic literacy [25], and a recent systematic review reached the same conclusion for nursing education [23]. The 2024 revision of the essential genomic nursing competencies opens by noting that workforce capacity remains low almost two decades after the competencies were first agreed [30]. Among physicians, a national survey of obstetricians in Singapore found high knowledge but wide variation in how NIPT was used [19], a Canadian survey found gaps in knowledge of cell-free DNA screening [20], and providers at a Nigerian tertiary hospital combined near-universal awareness with poor knowledge of complications [22]. Surveys of women in Romania and Iran found that informed choice was often not achieved [17,18], which fits with providers who struggle to explain the tests. What our data add is item-level detail from a system in which nurses carry most routine antenatal contacts. Nurses and midwives are the largest part of the health workforce worldwide [31], and they made up 82.5% of our sample.

### 4.2. Where the Knowledge Gaps Sit

Interpretation of combined-screening results was the weakest area. The test reports biochemical markers as multiples of the median, and the risk a woman is given depends on that transformation [7,8]. Only 16.4% of respondents identified MoM, and more than twice as many chose IU/L, the unit of raw hormone concentrations. A clinician who reads a MoM value as an absolute concentration cannot check a report for plausibility or explain it to a patient. Midwives and nurses do not calculate risk, but they are often the first to see the result (Figure 1), and what they need is to recognise what the number means and when to pass it on. Hospital and perinatal staff did better on this item than primary-care staff (36% versus 13% chose MoM; Supplementary Table S4).

The second-trimester item calls for a more cautious reading. The national standard specifies 19^+0^–21^+0^ weeks, while 18^+0^–21^+6^ weeks lies closer to international guidance for the anomaly scan [11]. With both answers accepted, two thirds answered correctly. Some of these answers probably reflect international or earlier national practice rather than an inability to time a scan, and the item should be revised so that only one answer is defensible.

The maternal-age item exposed an error in our answer key. We had keyed 35 years as correct, but the national orders use 37 years, and with combined screening, age is one term in an individual risk rather than a stand-alone indication [5,12]. Only 11.1% chose 37 years and 31.2% chose 40, which points to real uncertainty about when age should prompt referral. On the components of combined screening, only 36.3% chose ultrasound with PAPP-A and free β-hCG; answers that added alpha-fetoprotein or invasive testing suggest that the first- and second-trimester protocols are being mixed up.

### 4.3. Training, Reported Practice and Knowledge

Training attendance was associated with reported practice but not with knowledge. Part of the association is built into any practice score that counts attendance as a practice: with training as an item, the odds ratio was 9.86, and on the seven-item index it was 2.54. Trained respondents were also much more likely to call themselves fully confident in their knowledge (61.9% versus 27.6%; Supplementary Table S6), although they knew no more than untrained respondents.

These data cannot tell us why. Staff who choose to attend may already be more engaged with antenatal genetics, a selection effect that no cross-sectional design can remove. Furthermore, staff who have recently sat through a seminar may report more of what they were told is correct, and the practice block is entirely self-reported. Training in the region is delivered mainly as short seminars on procedures and referral pathways, which could change reported behaviour without correcting factual misconceptions, but we recorded nothing about the content, length or provider of the training each respondent attended. The Dutch experience shows that knowledge responds to training when it is measured: a mandatory blended-learning programme raised the proportion of correctly answered items among prenatal counsellors from 66% to 76% [21].

### 4.4. Self-Report and the NIPT Inconsistency

The same caution applies to the NIPT items. Only 11.0% listed NIPT among the methods they knew, yet 63.4% said they always recommend it when indicated, including 61.9% of those who had not mentioned it. Respondents may recognise the abbreviation in a directive question without being able to recall it unprompted, or the practice items may attract acquiescent answers. NIPT is not part of the guaranteed volume of free medical care in Kazakhstan and is available as a fee-based service, so “recommending” it may mean mentioning a private test rather than arranging one. Either way, self-reported practice is not a record of what happens in consultations, and the instrument needs validation against records or direct observation.

### 4.5. Organisational Barriers

The barriers respondents named were mostly structural. A shortage of specialists (42.6%) and distance (27.1%) fit the concentration of medical genetics in Almaty and Astana, and 13.0% did not know whether a genetics service operated in their area. Financial constraints (22.2%) suggest that parts of the pathway reach patients as paid services. Most respondents rated accessibility and the general public’s awareness of genetic services favourably, but 38.2% said that pregnant women are not given enough information. The two answers concern different groups, and the second is the more useful for planning: it points at the consultation rather than at public campaigns.

Reluctance to refer for invasive diagnosis was attributed mainly to procedure risk (53.3%) and patient fear (47.6%). The most recent meta-analysis puts the procedure-related risk of miscarriage at about 0.3% after amniocentesis and 0.2% after chorionic villus sampling, with confidence intervals that include no excess risk [32]. The perceived risk is therefore probably overstated and becomes a barrier in its own right. Cell-free DNA screening reduces the number of invasive procedures needed [9,10], which makes the low unprompted awareness of NIPT matter more than it might first appear.

### 4.6. Implications and Hypotheses for Implementation

These findings point to priorities; they do not show what will work, and we offer the following as hypotheses for implementation research. Training for primary-care nurses and midwives could be built around the items that failed: what a MoM value and a combined risk mean, the national ultrasound windows, the components of combined screening, and the national referral criteria, with age treated as one input into risk. Whether such training improves knowledge should be tested before and after the course, since attendance alone showed no relationship with knowledge here. Half of respondents relied on sources other than clinical guidelines, so access to current guidelines is a second target. Teleconsultation between primary care and the regional perinatal centre might ease the distance barrier, but it would need its own evaluation. Patient fear was the second most common reason for not referring, and one respondent in five could not say whether termination for a severe untreatable condition was appropriate. In a population in which families hold different views on testing and termination, counselling that respects informed choice belongs in any such programme alongside the facts.

### 4.7. Strengths and Limitations

The main strength of this study is its sample. With 1331 respondents drawn overwhelmingly from primary care and rural facilities, it reaches the part of the workforce that provider surveys usually miss, and it reports results at item level. The scoring rules and their arbitrary elements are stated explicitly (Table 1).

The limitations are substantial. The survey covered one region and used open recruitment, so a response rate cannot be calculated and the results cannot be generalised to Kazakhstan. Staff who felt more confident or more engaged with genetics may have been more willing to take part, which would bias knowledge upwards. The form was offered only in Kazakh, which may have excluded or disadvantaged staff who work mainly in Russian or Uzbek. Physicians were few, so their estimates are imprecise, particularly for the 15 other specialists. The questionnaire is new and has not undergone external content validation, test–retest assessment or confirmatory factor analysis. Its answer key contained an error (the maternal-age item), the respondent-level knowledge index and model still include that item, and one item has two defensible answers. Factorability was assessed on Pearson rather than tetrachoric or polychoric correlations. Several practice items describe physician tasks and had no “not applicable” option, so low practice scores among nurses partly reflect their role rather than the quality of their work. The dataset holds no respondent age, years of experience, facility or district; we could not adjust for these obvious confounders or model clustering, and the standard errors are probably too small. All responses were self-reported, the training question recorded neither content nor duration, and 19 records duplicating 13 response profiles could not be adjudicated. Finally, the design is cross-sectional, so none of the associations can be read as effects of training, awareness or attitudes.

## 5. Conclusions

Health workers in the Turkistan region strongly support prenatal screening and the development of genetic services, but their knowledge of the screening pathway does not match that support: on average about 55% of the knowledge items were answered correctly, and 14.6% of respondents reached the good-knowledge threshold. The gaps are concentrated in result interpretation, screening timing and the components of combined screening. Recent training was associated with better reported practice but not with better knowledge, and the design does not allow us to say whether training changes behaviour, attracts staff who are already engaged or changes how people report. Training for primary-care nurses and midwives, built around the items that failed, and evaluated by knowledge testing rather than attendance, is a reasonable next step. A repeat survey should record age, experience, facility and district, offer the form in Kazakh and Russian, and give a “not applicable” option for role-specific actions.

## Figures and Tables

**Table 1:** Items in the knowledge and practice indices and how they were scored.

Code	Item	Scoring
Knowledge index (12 items, 0–12; one point per correct answer)		
K1	Main purpose of prenatal genetic screening	Risk assessment for chromosomal anomalies
K2	Most important screening stages	First and second trimester
K3	Definition of karyotyping	Study of chromosome number and structure
K4	First-trimester serum markers used in Kazakhstan	PAPP-A and β-hCG
K5	Chromosome involved in Down syndrome	Chromosome 21
K6	Chromosome involved in Patau syndrome	Chromosome 13
K7	Chromosome involved in Edwards syndrome	Chromosome 18
K8	Timing of the first screening ultrasound	11^+0^–13^+6^ weeks
K9	Maternal age defining high genetic risk	35 years in the analysis key; the national orders specify 37 years ᵃ
K10	Timing of the second screening ultrasound	19^+0^–21^+0^ weeks ᵇ
K11	Components of combined first-trimester screening (multiple response)	Ultrasound and PAPP-A/β-hCG selected, no other option
K12	Unit in which combined-screening results are interpreted	MoM
Practice index (7 items, 0–14)		
P1	Recommends molecular genetic testing when indicated	Yes 2; sometimes 1; no 0
P2	Counsels couples planning pregnancy on inherited risk	Regularly 2; sometimes 1; no 0
P3	Recommends NIPT when indicated	Always 2; sometimes 1; never 0
P4	Discusses the importance of prenatal and neonatal screening	Frequently 2; sometimes 1; never 0
P5	Applies genetic test results in own practice	Regularly 2; sometimes 1; never 0
P6	Receives molecular genetic test results for patients	Frequently 2; rarely 1; never 0
P7	Referred patients to a medical geneticist in the past year	Frequently 2; sometimes 1; did not refer 0
Exposure (not part of either index)		
TR	Attended training or seminars on medical genetics in the last 3 years	Yes or no; counted as 2 or 0 only in the eight-item sensitivity version of the practice index

Note. ᵃ Kept in the respondent-level index as analysed; the mean of the 11 items without K9 is reported separately (Section 3.8). ᵇ 18^+0^–21^+6^ weeks also accepted in a sensitivity analysis of the mean. MoM, multiple of the median; NIPT, non-invasive prenatal testing; PAPP-A, pregnancy-associated plasma protein A; β-hCG, free β-human chorionic gonadotropin. Item codes match Supplementary Material S1.

**Table 2:** Characteristics of the 1331 respondents. Confidence intervals are Wilson intervals for a single proportion.

Characteristic	*n*	%	95% CI
Professional group			
Nurse	902	67.8	65.2–70.2
Midwife	196	14.7	12.9–16.7
General practitioner	159	11.9	10.3–13.8
Obstetrician-gynaecologist	59	4.4	3.5–5.7
Other specialist ^1^	15	1.1	0.7–1.9
Workplace			
Primary care ^2^	1146	86.1	84.1–87.9
Hospital	95	7.1	5.9–8.6
Perinatal care unit	90	6.8	5.5–8.2
Awareness of a local medical genetics service			
Fully aware	891	66.9	64.4–69.4
Partly aware	267	20.1	18.0–22.3
Not aware	173	13.0	11.3–14.9
Genetics training or seminar in the previous 3 years			
Yes	439	33.0	30.5–35.6
No	892	67.0	64.4–69.5
Main source of information about prenatal screening			
Clinical guidelines	667	50.1	47.4–52.8
Colleagues	253	19.0	17.0–21.2
Internet or social media	212	15.9	14.0–18.0
Scientific conferences or seminars	188	14.1	12.3–16.1
Other or uninterpretable	11	0.8	0.5–1.5

Note. ^1^ Neonatologists (*n* = 8), geneticists (*n* = 5) and ultrasound specialists (*n* = 2). ^2^ District and city polyclinics, primary health care centres and rural feldsher-midwife posts.

**Table 3:** Item-level performance on the 12 knowledge items, ordered by the proportion answering correctly (*n* = 1331).

Knowledge Item	Correct Answer	Correct *n* (%)	95% CI	Most Frequent Incorrect Answer, *n* (%)
Main purpose of prenatal genetic screening	Risk assessment for chromosomal anomalies	1224 (92.0)	90.4–93.3	Foetal sex determination, 55 (4.1)
Most important screening stages	First and second trimester	1195 (89.8)	88.0–91.3	Third trimester, 73 (5.5)
Definition of karyotyping	Study of chromosome number and structure	836 (62.8)	60.2–65.4	Method of detecting gene mutation, 243 (18.3)
First-trimester serum markers used in Kazakhstan	PAPP-A and β-hCG	776 (58.3)	55.6–60.9	PAPP-A, β-hCG and AFP, 478 (35.9)
Chromosome involved in Down syndrome	Chromosome 21	772 (58.0)	55.3–60.6	X chromosome, 269 (20.2)
Chromosome involved in Patau syndrome	Chromosome 13	758 (56.9)	54.3–59.6	Chromosome 18, 205 (15.4)
Chromosome involved in Edwards syndrome	Chromosome 18	686 (51.5)	48.9–54.2	Chromosome 13, 247 (18.6)
Timing of the first ultrasound screen	11^+0^–13^+6^ weeks	652 (49.0)	46.3–51.7	9^+0^–12^+6^ weeks, 350 (26.3)
Maternal age defining high genetic risk ᵃ	37 years (national orders) ᵃ	148 (11.1)	9.5–12.9	35 years, 550 (41.3)
Timing of the second ultrasound screen	19^+0^–21^+0^ weeks	483 (36.3)	33.7–38.9	18^+0^–21^+6^ weeks, 401 (30.1)
Components of combined first-trimester screening	Ultrasound plus PAPP-A and β-hCG only	483 (36.3)	33.7–38.9	Ultrasound alone, 292 (21.9)
Unit in which combined-screening results are interpreted	MoM	218 (16.4)	14.5–18.5	IU/L, 499 (37.5)

Note. AFP, alpha-fetoprotein; β-hCG, free β-human chorionic gonadotropin; MoM, multiple of the median; PAPP-A, pregnancy-associated plasma protein A. ᵃ Correct n shown for the national key (37 years). The analysis key used 35 years (550 correct, 41.3%); see Section 2.4.

**Table 4:** Responses to attitude and perception items (*n* = 1331). Items are reported individually; no composite score is calculated.

Attitude or Perception Item	Most Supportive or Most Confident, *n* (%)	Intermediate, *n* (%)	Least Supportive or Least Confident, *n* (%)
Referral of high-risk pregnancies to a geneticist	1162 (87.3)	144 (10.8)	25 (1.9)
Prenatal screening for all pregnant women	1126 (84.6)	176 (13.2)	29 (2.2)
Benefit of genetic counselling for reducing affected births	1169 (87.8)	4 (0.3)	158 (11.9)
Importance of developing national genetic services	974 (73.2)	298 (22.4)	59 (4.4)
Invasive diagnosis when clinically indicated ᵃ	1230 (92.5)	9 (0.7)	91 (6.8)
Self-rated confidence in prenatal genetics knowledge (self-assessment, reported separately)	517 (38.8)	768 (57.7)	46 (3.5)
Perceived accessibility of medical genetic care in own region (1 = not accessible to 5 = fully accessible)			
Rated 1 or 2	259 (19.5)		
Rated 3	208 (15.6)		
Rated 4 or 5	864 (64.9)		
Perceived level of public awareness of genetic services			
High	597 (44.9)		
Moderate	635 (47.7)		
Low	99 (7.4)		
Medically indicated termination for a severe untreatable inherited condition			
Considered appropriate	1029 (77.3)		
Unable to answer	254 (19.1)		
Not considered appropriate	48 (3.6)		

Note. For screening and referral, the most supportive option was “mandatory” and the intermediate option “recommended”; both are compatible with informed choice, which is why these items are not summed. Self-rated confidence is a self-assessment rather than an attitude and is shown separately. ᵃ Options were “necessary, mandatory” and “not necessary”; the intermediate column holds free-text answers (“with the woman’s consent”, “I do not know”); counts from the item-level export, *n* = 1330. Full response options are given in Supplementary Material S1.

**Table 5:** Self-reported clinical and counselling practice: the seven items of the practice index (*n* = 1331).

Practice Item	Consistent or Frequent (2), *n* (%)	Occasional (1), *n* (%)	Never or No (0), *n* (%)	Mean, % of Item Maximum
Recommends molecular genetic testing when indicated	985 (74.0)	245 (18.4)	101 (7.6)	83.2
Counsels couples planning pregnancy on inherited risk	934 (70.2)	309 (23.2)	88 (6.6)	81.8
Recommends NIPT when indicated	844 (63.4)	388 (29.2)	99 (7.4)	78.0
Discusses the importance of prenatal and neonatal screening	736 (55.3)	460 (34.6)	135 (10.1)	72.6
Applies genetic test results in own practice	641 (48.2)	477 (35.8)	213 (16.0)	66.1
Receives molecular genetic test results for patients	594 (44.6)	557 (41.8)	180 (13.5)	65.6
Referred patients to a medical geneticist in the past year	569 (42.7)	434 (32.6)	328 (24.6)	59.1

Note. NIPT, non-invasive prenatal testing. Attendance at genetics training in the previous three years (yes, 439; 33.0%) is analysed as an exposure and is not part of the practice index.

**Table 6:** Knowledge and practice indices by professional group, workplace and recent genetics training (*n* = 1331).

Group	n	Knowledge Index, Mean ± SD (0–12)	Practice Index, Mean ± SD ᵃ
Professional group			
Nurse	902	6.01 ± 2.59	10.14 ± 3.88 (0–16)
Midwife	196	7.33 ± 2.31	11.81 ± 3.12 (0–16)
General practitioner	159	6.97 ± 2.37	12.04 ± 2.93 (0–16)
Obstetrician-gynaecologist	59	9.20 ± 1.89	13.47 ± 2.47 (0–16)
Other specialist	15	8.20 ± 2.57	12.40 ± 2.59 (0–16)
Kruskal–Wallis test		H = 123.8; ε^2^ = 0.090	H = 91.4; ε^2^ = 0.066
Workplace			
Primary care	1146	6.46 ± 2.57	10.88 ± 3.65 (0–16)
Hospital	95	5.60 ± 2.71	8.82 ± 4.30 (0–16)
Perinatal care unit	90	7.77 ± 2.67	11.58 ± 3.62 (0–16)
Kruskal–Wallis test		H = 29.9; ε^2^ = 0.021	H = 24.8; ε^2^ = 0.017
Genetics training in the previous 3 years			
No	892	6.51 ± 2.57	9.39 ± 3.46 (0–14)
Yes	439	6.44 ± 2.71	11.62 ± 2.48 (0–14)
Mann–Whitney test		*p* = 0.522	*p* < 0.001, r = 0.39

Note. All Kruskal–Wallis tests *p* < 0.001. ε^2^, epsilon-squared (Kruskal–Wallis test); r, rank-biserial correlation (Mann–Whitney test). ^a^ By profession and workplace, practice is shown for the eight-item index, which includes 2 points for training attendance; the seven-item index was computed by profession and workplace only for the regression models. By training status, the seven-item index is shown.

**Table 7:** Multivariable logistic regression for good knowledge and good practice, with sandwich (HC1) standard errors (*n* = 1331). aOR, adjusted odds ratio; intervals that exclude 1 correspond to *p* < 0.05.

Predictor	Good Knowledge, 12-Item Index, aOR (95% CI)	Good Practice, 7-Item Index, aOR (95% CI)	Good Practice, 8-Item Index ^a^, aOR (95% CI)
Midwife vs. nurse	1.66 (1.08–2.56)	— ^b^	1.71 (1.14–2.58)
General practitioner vs. nurse	1.43 (0.89–2.29)	— ^b^	1.53 (1.01–2.34)
Obstetrician-gynaecologist vs. nurse	5.46 (2.93–10.19)	5.81 (2.86–11.82)	4.95 (2.48–9.89)
Other specialist vs. nurse	1.88 (0.56–6.32)	— ᵇ	3.25 (1.12–9.45)
Hospital vs. primary care	0.38 (0.16–0.88)	0.31 (0.16–0.59)	0.40 (0.21–0.77)
Perinatal care unit vs. primary care	1.27 (0.71–2.26)	0.23 (0.12–0.44)	0.30 (0.15–0.60)
Genetics training in previous 3 years	0.89 (0.63–1.26)	2.54 (1.90–3.40)	9.86 (7.10–13.69) ᵃ
Guidelines or conferences as main source	1.34 (0.94–1.92)	2.20 (1.66–2.93)	1.89 (1.38–2.59)
Awareness of genetic services, per level	1.54 (1.16–2.05)	— ^b^	1.52 (1.22–1.89)
Perceived accessibility, per point	1.24 (1.07–1.44)	— ^b^	1.32 (1.16–1.49)
Knowledge index, per point	n/a	— ^b^	1.02 (0.97–1.08)
Attitude score, per point ^c^	n/a	1.62 (1.47–1.78)	1.59 (1.44–1.76)
Events/n	194/1331	563/1331	522/1331
McFadden pseudo-R^2^	0.082	— ^b^	0.330

Note. All models also include the variables listed in Section 2.5. ^a^ Training attendance is both a predictor and an item of the eight-item index, so its estimate in that column is inflated by construction. ^b^ Not reported in the analysis output for this model, which described these terms as unchanged in direction and similar in magnitude to the eight-item model. ^c^ Former summed attitude score (0–12), retained as a covariate in the practice models; see Section 2.5.

**Table 8:** Sensitivity analyses.

Analysis	Knowledge	Practice
Index as analysed, mean ± SD (% of maximum)	6.49 ± 2.62 of 12 (54.1)	10.12 ± 3.33 of 14 (72.3)
Maternal-age item removed, mean (% of maximum) ^a^	6.07 of 11 (55.2)	n/a
Maternal-age item removed and 18^+0^–21^+6^ weeks also accepted, mean (%) ^a^	6.37 of 11 (57.9)	n/a
Training counted as a practice item, mean ± SD (%)	n/a	10.78 ± 3.74 of 16 (67.4)
Repeated response profiles removed (n = 1312), mean % of maximum	53.9 (12 items)	67.3 (8 items)
Good category, n (%)	194 (14.6)	563 (42.3); with training as an item 522 (39.2)
aOR for recent training (95% CI)	0.89 (0.63–1.26)	2.54 (1.90–3.40); with training as an item 9.86 (7.10–13.69)

Note. aOR, adjusted odds ratio. ^a^ Exact: the mean of an additive index equals the sum of its item proportions, computed here from the item counts in Table 3.

## Data Availability

The anonymized item-level data are available from the corresponding authors on reasonable request, subject to approval by the Local Bioethics Commission of Khoja Akhmet Yassawi International Kazakh-Turkish University. The survey was anonymous and collected no names, contact details, facility identifiers or clinical records.

## Supplementary Materials

The following supporting information can be downloaded at: https://www.mdpi.com/article/10.3390/healthcare14213776/s1, Supplementary Material S1: Questionnaire (Kazakh original and English translation) with item codes, response distributions and scoring key; Supplementary Material S2: Ethics conclusion and KazREC certificate; Table S1: Multiple-response questions, overall and by level of care; Table S2: CHERRIES checklist; Table S3: Knowledge items by level of care; Table S4: Item responses by level of care; Table S5: Former attitude composite; Table S6: Selected exploratory cross-tabulations; Figure S1: Location of Turkistan region relative to the national medical genetics services; Figure S2: Directed acyclic graph for the practice models.
