# Supplementary material for: Knowledge, Attitudes and Practices Regarding Prenatal Screening and Genetic Diagnosis of Chromosomal Anomalies Among Health Workers in Turkistan Region, Kazakhstan: A Cross-Sectional Survey of a Predominantly Primary-Care Workforce
Source: Healthcare (Basel). 2026 Nov 5;14(21):3776. doi: 10.3390/healthcare14213776 (PMC13762700; doi:10.3390/healthcare14213776)
Supplement: Supplementary file 1 [file healthcare-14-03776-s001.zip › healthcare-4613524-supplementary.pdf]

Supplementary Materials

Knowledge, Attitudes and Practices Regarding Prenatal Screening and Genetic Diagnosis of Chromosomal Anomalies Among Health Workers in Turkistan Region, Kazakhstan: A Cross-Sectional Survey of a Predominantly Primary-Care Workforce

Zhansaya Torgauytova, Ardak Ayazbekov, Sundetgali Kalmakhanov, Almagul Kurmanova, Seçil Özkan, Saken Khaidarov, Gaukhar Kurmanova, Arman Nurmakhanbetov, Aknur Turgumbayeva, Amina Abdrakhmanova, Diana Sundetova, Shynar Zakaryanova and Elnur Adilova

Healthcare (MDPI), manuscript healthcare-4613524, revised version R1

Contents

- Supplementary Material S1. Questionnaire (Kazakh original and English translation) with item codes, response distributions and scoring key
- Supplementary Material S2. Ethics conclusion and KazREC certificate (separate file, unchanged)
- Table S1. Multiple-response questions, overall and by level of care
- Table S2. CHERRIES checklist
- Table S3. Knowledge items by level of care
- Table S4. Item responses by level of care
- Table S5. Former attitude composite
- Table S6. Selected exploratory cross-tabulations
- Figure S1. Location of Turkistan region relative to the national medical genetics services
- Figure S2. Directed acyclic graph for the practice models

Note on denominators. Item-level tallies in S1 and Tables S1, S3 (by level of care), S4 and S6 come from the institutional cross-tabulation export, which holds 1330 records (1150 primary care, 180 hospital or perinatal unit). The analysis file used in the manuscript holds 1331 records (1146 primary care, 95 hospital, and 90 perinatal unit). The two files were exported separately and differ by one record and in how the five records are grouped by workplace. Percentages here use the export denominators; the manuscript uses 1331.

Supplementary Material S1. Questionnaire and scoring key

S1.1. Structure and administration

The questionnaire was written by the study team in Kazakh and administered online through Google Forms between 1 June and 30 July 2026. It has 38 items: 35 content items in four blocks, reproduced in full below, and three respondent items (consent, profession and workplace; S1.2). By domain, the content items comprise 14 knowledge items (11 single-choice and 3 multiple-response), 11 attitude and perception items, and 10 practice items. Five content questions allowed several answers: regulatory documents (KD1), diagnostic methods (KD2), components of combined screening (K11), barriers (BR), and reasons for not referring for invasive diagnosis (RR). All items were mandatory. Free-text answers were possible for some items; they are listed as "free text" below. The English translation was prepared for publication by Z.T. and back-translated by an independent bilingual public health specialist.

S1.2. Respondent items

Item	Question	Response options	n (%)
C1	Consent to take part	Yes (required to proceed)	1331 (100)
R1	Profession	Nurse / midwife / general practitioner / obstetrician-gynecologist / other specialist (neonatologist, geneticist, ultrasound specialist)	902 / 196 / 159 / 59 / 15
R2	Workplace	Primary care (polyclinic, primary health care centre, feldsher-midwife post) / hospital / perinatal care unit	1146 / 95 / 90

Counts from the analysis file (n = 1331).

S1.3. Content items with response distribution and scoring key

For each item the Kazakh original is followed by the English translation. Counts are from the institutional export (n = 1330). Score column: 1/0, knowledge key; 2/1/0, ordinal coding (practice items are summed into the 7-item practice score; attitude items are reported individually and no longer summed); ✓/✗, options required/excluded for a correct answer on a multiple-response item; R/D, relevant document/distractor; —, not scored.

Block 1. Awareness and knowledge of prenatal screening

Code	Kazakh original	English translation	n (%)	Score
**AW · Perception · single choice**				
	Өзіңіз қызмет атқаратын өңірде медико-генетикалық көмек көрсету қызметінің бар екенін білесіз бе?	Do you know whether a medical genetics service operates in the region where you work?		
	Иә, толық	Yes, fully	890 (66.9)	2
	Ішінара	Partly	267 (20.1)	1
	Хабарым жоқ	Not aware	173 (13.0)	0
		Note: Covariate (awareness, 0–2); not part of any score.		
**KD1 · Knowledge (descriptive) · multiple response**				
	Қазақстан Республикасында жүктілік кезіндегі пренаталды скринингті реттейтін негізгі нормативтік құжаттар қандай?	Which are the main regulatory documents governing prenatal screening during pregnancy in the Republic of Kazakhstan?		
	ҚР ДСМ № 272 бұйрығы, 27 сәуір 2015 ж. «Денсаулық сақтау саласындағы мемлекеттік көрсетілетін қызметтер стандарттарын бекіту туралы»	Order No. 272 of 27 April 2015 on state service standards in health care (distractor)	81 (6.1)	D
	ҚР ДСМ № 52 бұйрығы, 31 наурыз 2023 – неонатальды көмек көрсету стандарттары	Order No. 52 of 31 March 2023, standards of neonatal care (distractor)	169 (12.7)	D
	ҚР ДСМ № ҚР ДСМ-54 (2022) «Стандартты пренаталды ультрадыбыстық скрининг жүргізу	Order No. ҚR DSM-54 (2022), standard for organizing prenatal ultrasound screening	397 (29.8)	R

Code	Kazakh original	English translation	n (%)	Score
	ұйымының стандарты»			
	ҚР ДСМ № 704 бұйрығы(2010) «Скринингті ұйымдастыру қағидаларын бекіту туралы»	Order No. 704 (2010), rules for organizing screening	406 (30.5)	R
	ҚР ДСМ № 92 бұйрығы (2021 жылғы 26 тамыз) «Қазақстан Республикасында акушерлік-гинекологиялық көмекті ұйымдастыру стандарты туралы»	Order No. ҚR DSM-92 (26 August 2021), standard for organizing obstetric and gynecological care	934 (70.2)	R
		Note: Descriptive only. R, relevant document; D, distractor.		
**KD2 · Knowledge (descriptive) · multiple response**				
	Сіз жүктілік кезінде жасалатын қандай генетикалық диагностика әдістерін білесіз?	Which genetic diagnostic methods used during pregnancy do you know?		
	Пренатальды ультрадыбыстық (УДЗ) скрининг	Prenatal ultrasound screening	1027 (77.2)	✓
	Биохимиялық скрининг (PAPP-A, β-ХГЧ және т.б.)	Biochemical screening (PAPP-A, β-hCG, etc.)	813 (61.1)	✓
	Инвазивті емес пренатальды тестілеу (НИПТ)	Non-invasive prenatal testing (NIPT)	147 (11.1)	✓
	Инвазивті диагностика (амниоцентез, хорион биопсиясы)	Invasive diagnosis (amniocentesis, chorionic villus sampling)	295 (22.2)	✓
	Молекулалық-генетикалық тесттер	Molecular genetic tests	364 (27.4)	✓
	Білмеймін	I do not know	30 (2.3)	—
		Note: Descriptive only (number of methods recognized, 0–5).		
**K1 · Knowledge · single choice**				
	Пренаталды генетикалық скринингтің негізгі мақсаттары қандай?	What are the main aims of prenatal genetic screening?		
	Хромосомалық ақаулардың даму қаупін анықтау	Assessing the risk of chromosomal anomalies	1223 (92.0)	1
	Нәрестенің жынысын анықтау	Determining fetal sex	55 (4.1)	0
	Плацента қызметін бағалау	Assessing placental function	52 (3.9)	0
**K8 · Knowledge · single choice**				
	Жүктілік кезінде бірінші пренаталды ультрадыбыстық скрининг жүктіліктің қай аптасында жасалады?	In which week of pregnancy is the first prenatal ultrasound screening performed?		
	9+0-12+6 аптада	9^{+0}–12^{+6} weeks	350 (26.3)	0
	10+0-14+6 аптада	10^{+0}–14^{+6} weeks	137 (10.3)	0
	11+0-13+6 аптада	11^{+0}–13^{+6} weeks	651 (48.9)	1
	12+0-14+6 аптада	12^{+0}–14^{+6} weeks	192 (14.4)	0
		Note: Keyed to Order No. ҚR DSM-54.		
**K10 · Knowledge · single choice**				
	Жүктілік кезінде екінші пренаталды ультрадыбыстық скрининг жүктіліктің қай аптасында жасалады?	In which week of pregnancy is the second prenatal ultrasound screening performed?		
	16+0-20+0 аптада	16^{+0}–20^{+0} weeks	215 (16.2)	0
	18+0-21+6 аптада	18^{+0}–21^{+6} weeks	401 (30.2)	0 (1 in sensitivity analysis)
	19+0-21+0 аптада	19^{+0}–21^{+0} weeks	483 (36.3)	1
	19+6-21+0 аптада	19^{+6}–21^{+0} weeks	231 (17.4)	0
		Note: Keyed to Order No. ҚR DSM-54; 18^{+0}–21^{+6}		

Code	Kazakh original	English translation	n (%)	Score
		weeks also accepted in a sensitivity analysis.		
**K4 · Knowledge · single choice**				
	Жүктілік кезіндегі даму ақаулары және хромосомдық ауытқуларды анықтауда1-ші триместірінде жасалатын ана қаны сарысуының (МСМ) биохимиялық маркерлерінің қай түрлері ҚР қолданылады	Which first-trimester maternal serum biochemical markers are used in Kazakhstan to detect developmental defects and chromosomal anomalies?		
	PAPP-A, β-ХГЧ	PAPP-A, β-hCG	776 (58.3)	1
	PAPP-A, β-ХГЧ, АФП	PAPP-A, β-hCG, AFP	477 (35.9)	0
	Ингибин А	Inhibin A	26 (2.0)	0
	Конъюгирленбеген эстрадиол	Unconjugated estradiol	51 (3.8)	0
**K11 · Knowledge · multiple response**				
	Жүктіліктің бірінші триместрінде жүргізілетін комбинирленген пренаталды скрининг қандай зерттеулерден тұрады?	Which examinations make up combined prenatal screening in the first trimester?		
	Ультрадыбыстық зерттеу	Ultrasound examination	1042 (78.3)	✓
	Биохимиялық маркерлерді анықтау (PAPP-A, β-ХГЧ)	Biochemical markers (PAPP-A, β-hCG)	930 (69.9)	✓
	Инвазивті тексеру (амниоцентез)	Invasive testing (amniocentesis)	153 (11.5)	✗
	Конъюгирленбеген эстриол	Unconjugated estriol	22 (1.7)	✗
	АФП және ингибин А	AFP and inhibin A	50 (3.8)	✗
	Жүктілікті визуалды бақылау	Visual follow-up of the pregnancy	189 (14.2)	✗
		Note: Multiple response. Scored 1 only if both ✓ options and no ✗ option were selected (n = 483, 36.3%).		
**K3 · Knowledge · single choice**				
	Кариотиптеу дегеніміз не?	What is karyotyping?		
	Хромосомалар саны мен құрылымын зерттеу әдісі	A method for studying chromosome number and structure	835 (62.8)	1
	Геннің мутациясын анықтау әдісі	A method for detecting gene mutations	243 (18.3)	0
	УДЗ арқылы ұрықтың даму кезеңін бағалау	Assessing fetal developmental stage by ultrasound	191 (14.4)	0
	Ағзадағы гормон деңгейін бағалау	Assessing hormone levels in the body	61 (4.6)	0
**K9 · Knowledge · single choice**				
	Жүкті әйелдер қай жастан туа біткен және тұқым қуалайтын патологияның дамуы бойынша жоғары қаупі тобына жатады?	From what age are pregnant women considered at high risk of congenital and hereditary pathology?		
	30 жастан бастап	From 30 years	181 (13.6)	—
	32 жастан бастап	From 32 years	36 (2.7)	—
	35 жастан бастап	From 35 years	550 (41.4)	1 (analysis key)
	37 жастан бастап	From 37 years	148 (11.1)	National orders
	40 жастан бастап	From 40 years	415 (31.2)	—
		Note: Keyed to 35 years in the analysis; Orders No. 704 and No. ҚR DSM-92 use 37 years, and in combined screening age is one input into individual risk (see S1.5).		
**K12 · Knowledge · single choice**				
	Жүктіліктің1-ші триместіріндегі пренаталдық	In what unit are the results of first-trimester		

Code	Kazakh original	English translation	n (%)	Score
	(комбинирленген) скрининг нәтижелері қандай бірлікпен интерпретацияланады?	(combined) prenatal screening interpreted?		
	MoM	MoM (multiple of the median)	218 (16.4)	1
	МЕ/л	IU/L	499 (37.5)	0
	г/л	g/L	290 (21.8)	0
	ММ	"MM" (abbreviation as printed on the form)	323 (24.3)	0

Block 2. Attitudes and perceptions

Code	Kazakh original	English translation	n (%)	Score
**A2 · Attitude · single choice**				
	Сіз барлық жүкті әйелдерге ұрықтың даму ақауы және хромосомдық аномалияны анықтау үшін пренатальды скринингті міндетті түрде жүргізу қажет деп ойлайсыз ба?	Do you think prenatal screening for fetal malformations and chromosomal anomalies should be mandatory for all pregnant women?		
	Иә, міндетті түрде	Yes, mandatory	1126 (84.7)	2
	Иә, ұсынылады	Yes, recommended	109 (8.2)	1
	Тек көрсеткіштер бойынша	Only when indicated	66 (5.0)	1
	Жоқ	No	29 (2.2)	0
		Note: Reported individually; former composite coding shown.		
**A1 · Attitude · single choice**				
	Туа біткен және тұқым қуалайтын патология бойынша «қауіп тобына» жататын жүкті әйелдерді генетик дәрігерге жолдауға көзқарасыңыз	What is your attitude to referring pregnant women at risk of congenital and hereditary pathology to a geneticist?		
	Міндетті деп есептеймін	I consider it mandatory	1161 (87.3)	2
	Ұсынылады, бірақ міндетті емес	Recommended but not mandatory	106 (8.0)	1
	Қажеті жоқ, ерікті түрде болу керек	Not needed, should be voluntary	38 (2.9)	1
	Теріс қараймын	Negative	25 (1.9)	0
		Note: Reported individually; former composite coding shown.		
**TOP · Perception · single choice**				
	Сіздің пікіріңізше, ауыр емдеуге келмейтін тұқым қуалайтын аурулар анықталған жағдайда медициналық көрсеткіштер бойынша жүктілікті үзу дұрыс па?	In your opinion, is termination of pregnancy on medical grounds appropriate when a severe untreatable hereditary disease is detected?		
	Иә	Yes	1029 (77.4)	—
	Жауап беруге қиналамын	Difficult to answer	253 (19.0)	—
	Жоқ	No	48 (3.6)	—
		Note: Reported descriptively; never scored (ethical position).		
**PAW · Perception · single choice**				
	Халықтың медико-генетикалық қызмет мүмкіндіктері туралы ақпараттану деңгейін қалай бағалайсыз?	How do you rate the population's awareness of what medical genetics services offer?		
	Жоғары	High	597 (44.9)	—
	Орташа	Moderate	634 (47.7)	—
	Төмен	Low	99 (7.4)	—

Code	Kazakh original	English translation	n (%)	Score
		Note: Descriptive only.		
**AC · Perception · single choice**				
	Сіз өз өңіріңізде жүкті әйелдерге медико-генетикалық көмектің қолжетімділігін қалай бағалайсыз? (1-ден 5-ке дейінгі шкала бойынша бағалаңыз)	How do you rate the accessibility of medical genetic care for pregnant women in your region? (scale 1–5)		
	1 – Қол жетімді емес	1 – Not accessible	149 (11.2)	1
	2 – Қиындықпен қолжетімді	2 – Accessible with difficulty	110 (8.3)	2
	3 – Ішінара қолжетімді	3 – Partly accessible	207 (15.6)	3
	4 – Жалпы қолжетімді	4 – Generally accessible	496 (37.3)	4
	5 – Толығымен қолжетімді	5 – Fully accessible	368 (27.7)	5
		Note: Covariate (1–5).		
**BR · Perception · multiple response**				
	Жүкті әйелдердің генетикалық көмек алуындағы негізгі кедергілер қандай?	What are the main barriers to pregnant women receiving genetic care?		
	Мамандардың жоқтығы	Shortage of specialists	567 (42.6)	—
	Жүкті әйелдердің жеткіліксіз ақпараттандырылуы	Inadequate information for pregnant women	508 (38.2)	—
	Географиялық алшақтық	Geographical distance	361 (27.1)	—
	Қаржылық шектеулер (қызметтер тек ақылы)	Financial constraints (services are fee-based only)	294 (22.1)	—
	Медицина қызметкерлерінің жеткіліксіз хабардар болуы	Insufficient awareness among health workers	160 (12.0)	—
		Note: Multiple response; descriptive.		
**A5 · Attitude · single choice**				
	Сіздің пікіріңізше көрсеткіш болған жағдайда инвазивті пренаталды диагностика жасау қажет пе?	In your opinion, is invasive prenatal diagnosis necessary when indicated?		
	Қажет, міндетті түрде	Necessary, mandatory	1230 (92.5)	2
	Жүктінің келісуімен	With the pregnant woman's consent (free text)	3 (0.2)	—
	Қажеті жоқ	Not necessary	91 (6.8)	0
	Билмеймин	I do not know (free text)	6 (0.5)	0
		Note: Reported individually (main text, Table 4).		
**A4 · Attitude · single choice**				
	Медициналық генетика саласындағы қызметтерді дамыту еліміз үшін маңызды деп ойлайсыз ба?	Do you think developing medical genetics services is important for the country?		
	Иә, өте маңызды	Yes, very important	973 (73.2)	2
	Иә, маңызды	Yes, important	298 (22.4)	1
	Жауап беру қиын	Difficult to answer	46 (3.5)	0
	Жоқ, маңызды емес	No, not important	13 (1.0)	0
		Note: Reported individually; former composite coding shown.		
**A6 · Self-assessment · single choice**				
	Жүктілік кезіндегі ұрықтың даму ақаулары және хромосмдық аномалияларын анықтау барысындағы пренатальды генетикалық	How confident are you in your knowledge of prenatal genetic screening for fetal malformations and chromosomal anomalies?		

Code	Kazakh original	English translation	n (%)	Score
	скрининг бойынша өз біліміңізге қаншалықты сенімдісіз?			
	Толығымен сенімдімін	Fully confident	517 (38.9)	2
	Негізгі аспектілерін білемін	I know the main aspects	251 (18.9)	1
	Қосымша білім қажет	I need further training	516 (38.8)	1
	Тақырыпты түсінбеймін	I do not understand the topic	46 (3.5)	0
		Note: Self-assessment; reported separately.		
**A3 · Attitude · single choice**				
	Генетикалық кеңес берудің енгізілуі тұқым қуалайтын аурулары бар балалардың туылу жиілігін төмендетеді деп ойлайсыз ба?	Do you think introducing genetic counselling reduces the birth rate of children with hereditary diseases?		
	Иә	Yes	1169 (87.9)	2
	Мумкін	Maybe (free text)	8 (0.6)	1
	Жоқ	No	152 (11.4)	0
	Түсінбедім	Did not understand (free text)	1 (0.1)	0
		Note: Offered options were yes and no; other answers are free text.		

Block 3. Clinical and counselling practice

Code	Kazakh original	English translation	n (%)	Score
**P7 · Practice · single choice**				
	Соңғы бір жыл ішінде пациенттерді медициналық генетик дәрігеріне консультацияға жолдадыңызба?	In the past year, have you referred patients to a medical geneticist for consultation?		
	Жиі	Frequently	569 (42.8)	2
	Кейде	Sometimes	434 (32.6)	1
	Жолдамадым	Did not refer	327 (24.6)	0
		Note: Physician task under the national orders.		
**P5 · Practice · single choice**				
	Медико-генетикалық зерттеулердің нәтижелерін өз практикаңызда қолданасыз ба?	Do you use the results of medical genetic tests in your practice?		
	Иә, тұрақты түрде	Yes, regularly	641 (48.2)	2
	Кейде	Sometimes	477 (35.9)	1
	Ешқашан	Never	212 (15.9)	0
		Note: Physician task under the national orders.		
**P4 · Practice · single choice**				
	Пренатальды және неонатальды скринингтің маңыздылығы туралы пациенттермен әңгіме өткізесіз бе?	Do you talk with patients about the importance of prenatal and neonatal screening?		
	Жиі	Frequently	736 (55.3)	2
	Кейде	Sometimes	460 (34.6)	1
	Ешқашан	Never	131 (9.8)	0
	Әрине	Of course (free text)	3 (0.2)	0*
		Note: *Free-text answer coded 0 under the conservative rule.		
**P1 · Practice · single choice**				

Code	Kazakh original	English translation	n (%)	Score
	Пациенттерге (көрсеткіштер бойынша молекулярлы-генетикалық тестілеуден өтуге ұсыныс бересізбе?	Do you recommend molecular genetic testing to patients when indicated?		
	Иә	Yes	985 (74.1)	2
	Кейде	Sometimes	245 (18.4)	1
	Жоқ	No	100 (7.5)	0
		Note: Physician task under the national orders.		
**SRC · Practice (descriptive) · single choice**				
	Жүктілік кезіндегі пренаталдық скрининг туралы ақпаратты қайдан аласыз?	Where do you get information about prenatal screening during pregnancy?		
	Клиникалық нұсқаулықтар	Clinical guidelines	667 (50.2)	EBM
	Ғылыми конференциялар / семинарлар	Scientific conferences or seminars	188 (14.1)	EBM
	Әріптестерден	Colleagues	253 (19.0)	—
	Интернет/әлеуметтік желілер	Internet or social media	212 (15.9)	—
	Әрдайым	"Always" (free text)	6 (0.5)	Other
	Кейде	"Sometimes" (free text)	2 (0.2)	Other
	Поликлиника	"Polyclinic" (free text)	1 (0.1)	Other
	Уч.мейірбикеден	"From the district nurse" (free text)	1 (0.1)	Other
		Note: Covariate: guidelines or conferences (EBM) versus other.		
**P2 · Practice · single choice**				
	Жүктілікті жоспарлап жүрген жұптарға тұқым қуалайтын аурулардың қаупі туралы әңгіме жүргізесіз бе?	Do you talk with couples planning a pregnancy about the risk of hereditary diseases?		
	Иә, тұрақты түрде	Yes, regularly	933 (70.2)	2
	Кейде	Sometimes	309 (23.2)	1
	Жоқ	No	88 (6.6)	0
**P6 · Practice · single choice**				
	Пациенттердің молекулярлы-генетикалық тест нәтижелерін (мысалы, тұқым қуалайтын ауруларға күдіктенген жағдайда қаншалықты жиі аласыз?	How often do you receive patients' molecular genetic test results (e.g., when a hereditary disease is suspected)?		
	Жиі	Frequently	594 (44.7)	2
	Сирек	Rarely	556 (41.8)	1
	Ешқашан	Never	180 (13.5)	0
		Note: Physician task under the national orders.		
**P3 · Practice · single choice**				
	Көрсеткіш болған жағдайда пациенттерге НИПТ (инвазивті емес пренатальды тестілеу) өтуге кеңес бересізбе?	Do you advise patients to undergo NIPT (non-invasive prenatal testing) when indicated?		
	Иә, әрдайым	Yes, always	844 (63.5)	2
	Кейде	Sometimes	387 (29.1)	1
	Ешқашан	Never	99 (7.4)	0
		Note: Physician task under the national orders.		
**TR · Exposure · single choice**				
	Соңғы 3 жылда медициналық генетика бойынша оқу немесе семинарларға	Have you attended training or seminars on medical genetics in the last 3 years?		

Code	Kazakh original	English translation	n (%)	Score
	қатыстыңыз ба?			
	Иә	Yes	438 (32.9)	1
	Жоқ	No	892 (67.1)	0
		Note: Exposure; counted as 2/0 only in the eight-item sensitivity version of the practice index.		

Block 4. Cytogenetics, screening stages and invasive diagnosis

Code	Kazakh original	English translation	n (%)	Score
**K5 · Knowledge · single choice**				
	Трисомиялар (Даун синдромы) қай хромосоманың аномалиясымен байланысты?	Trisomy (Down syndrome) is associated with an anomaly of which chromosome?		
	21-хромосома	Chromosome 21	772 (58.0)	1
	13-хромосома	Chromosome 13	205 (15.4)	0
	18-хромосома	Chromosome 18	81 (6.1)	0
	Х-хромосома	X chromosome	269 (20.2)	0
	Жауап жоқ	No answer	3 (0.2)	0
**K7 · Knowledge · single choice**				
	Трисомиялар (Эдвардс синдромы) қай хромосомалардың аномалиясымен байланысты?	Trisomy (Edwards syndrome) is associated with an anomaly of which chromosome?		
	18-хромосома	Chromosome 18	686 (51.6)	1
	13-хромосома	Chromosome 13	247 (18.6)	0
	21-хромосома	Chromosome 21	199 (15.0)	0
	Х-хромосома	X chromosome	194 (14.6)	0
	Жауап жоқ	No answer	4 (0.3)	0
**K6 · Knowledge · single choice**				
	Трисомиялар (Патау синдромы) қай хромосомалардың аномалиясымен байланысты?	Trisomy (Patau syndrome) is associated with an anomaly of which chromosome?		
	13-хромосома	Chromosome 13	758 (57.0)	1
	18-хромосома	Chromosome 18	205 (15.4)	0
	21-хромосома	Chromosome 21	190 (14.3)	0
	Х-хромосома	X chromosome	173 (13.0)	0
	Жауап жоқ	No answer	4 (0.3)	0
**K2 · Knowledge · single choice**				
	Жүктілік кезінде жасалатын пренаталды скринингтің қай кезеңдері ең маңызды?	Which stages of prenatal screening during pregnancy are the most important?		
	1-ші және 2-ші триместр	First and second trimester	1194 (89.8)	1
	3-ші триместр	Third trimester	73 (5.5)	0
	Тек босану алдында	Only before delivery	27 (2.0)	0
	Жүктілік кезінде скринингҚажет емес	Screening is not needed during pregnancy	36 (2.7)	0
**RR · Practice (descriptive) · multiple response**				
	Сіздің жүкті әйелдерді пренаталды инвазивті диагностика жасауға (мысалы, амниоцентез-қағанақ суын алып зерттеу) жолдаудан бас	What are your reasons for not referring pregnant women for invasive prenatal diagnosis (e.g., amniocentesis)?		

Code	Kazakh original	English translation	n (%)	Score
	тартуыңыздың себебі?			
	Инвазивті әдістердің асқыну қаупінің болуы (жүктіліктің үзілуі, инфекция т.б.)	Risk of complications (pregnancy loss, infection, etc.)	709 (53.3)	—
	Пациенттердің қорқынышы және инвазивті процедураны қабылдамауы	Patients' fear and refusal of the procedure	634 (47.7)	—
	Медициналық этика немесе құқықтық мәселелер (рұқсат алу қиындығы)	Ethical or legal issues (difficulty obtaining permission)	181 (13.6)	—
	Пациенттің әлеуметтік-экономикалық жағдайы (қаржылық мүмкіндіктері жоқ)	Patient's socioeconomic situation (cannot afford it)	164 (12.3)	—
	Құрал-жабдықтардың немесе мамандардың жетіспеушілігі	Lack of equipment or specialists	150 (11.3)	—
	Инвазивті диагностика нәтижелерін өңдеу мен түсіндіру қиындықтары	Difficulty processing and interpreting results	146 (11.0)	—
	Медицина қызметкерлерінің тәжірибесінің жеткіліксіздігі ,білім деңгейінің төмендігі	Insufficient experience or knowledge of health workers	108 (8.1)	—
	Басқа себептер	Other reasons	134 (10.1)	—
		Note: Multiple response; descriptive.		

S1.4. Scoring rules

Knowledge index (0–12). One point was awarded for each correct answer on K1–K12. K11 (multiple response) scores 1 only when ultrasound and the PAPP-A/β-hCG option are both selected and no other option is selected. K9 (maternal age) was keyed to 35 years in the analysis; the national orders specify 37 years. The mean of the 11 items without K9, and with 18^{+0}–21^{+6} weeks also accepted for K10, is reported in the main text (Table 8).

Practice index (0–14). This is the sum of P1–P7 coded 2/1/0. Training attendance (TR) is analyzed as an exposure, not a practice; an eight-item version counting TR as 2/0 is used in a sensitivity analysis.

Attitude items. A1–A5 are reported individually; A6 (self-rated confidence) is a self-assessment and is reported separately. The previously summed attitude score (A1–A6, 0–12) is described in Table S5 and is used only as a covariate in the practice models.

Categories. For knowledge and practice: a score below 60% of the maximum, poor; 60–79.9% is moderate; 80% or above is good. These are operational cut-points, not validated competency standards.

Free text. Free-text answers that matched a predefined category were recoded to it; others were coded as 0 (practice) or "other" (information source).

S1.5. Notes on the key

• K9: the analysis key (35 years) did not match Orders No. 704 and No. ҚR DSM-92 (37 years). This is an error in the answer key, not in data collection; the item and its options were presented as designed.
• K11: a multiple-response item, scored all-or-nothing as described in S1.4.
• K12: the question asked how results are interpreted.
• TR: this is not part of the practice index.
• Attitude items: these are not summed, because the coding ranked "mandatory" above "recommended", A6 measures confidence, and the sum had a ceiling effect.

Table S1. Multiple-response questions, overall and by level of care

Response	Overall, n (%) N = 1330	95% CI	Primary care, n (%) N = 1150	Hospital or perinatal, n (%) N = 180	p
**KD1. Which are the main regulatory documents governing prenatal screening during pregnancy in the Republic of Kazakhstan?**					
Order No. 272 of 27 April 2015 on state service standards in health care (distractor)	81 (6.1)	4.9–7.5	58 (5.0)	23 (12.8)	<0.001
Order No. 52 of 31 March 2023, standards of neonatal care (distractor)	169 (12.7)	11.0–14.6	149 (13.0)	20 (11.1)	0.549
Order No. ҚR DSM-54 (2022), standard for organizing prenatal ultrasound screening	397 (29.8)	27.5–32.4	326 (28.3)	71 (39.4)	0.004
Order No. 704 (2010), rules for organizing screening	406 (30.5)	28.1–33.1	317 (27.6)	89 (49.4)	<0.001
Order No. ҚR DSM-92 (26 August 2021), standard for organizing obstetric and gynecological care	934 (70.2)	67.7–72.6	820 (71.3)	114 (63.3)	0.035
**KD2. Which genetic diagnostic methods used during pregnancy do you know?**					
Prenatal ultrasound screening	1027 (77.2)	74.9–79.4	874 (76.0)	153 (85.0)	0.007
Biochemical screening (PAPP-A, β-hCG, etc.)	813 (61.1)	58.5–63.7	701 (61.0)	112 (62.2)	0.805
Non-invasive prenatal testing (NIPT)	147 (11.1)	9.5–12.9	108 (9.4)	39 (21.7)	<0.001
Invasive diagnosis (amniocentesis, chorionic villus sampling)	295 (22.2)	20.0–24.5	228 (19.8)	67 (37.2)	<0.001
Molecular genetic tests	364 (27.4)	25.0–29.8	308 (26.8)	56 (31.1)	0.243
I do not know	30 (2.3)	1.6–3.2	25 (2.2)	5 (2.8)	0.588
**K11. Which examinations make up combined prenatal screening in the first trimester?**					
Ultrasound examination	1042 (78.3)	76.1–80.5	901 (78.3)	141 (78.3)	>0.999
Biochemical markers (PAPP-A, β-hCG)	930 (69.9)	67.4–72.3	802 (69.7)	128 (71.1)	0.793
Invasive testing (amniocentesis)	153 (11.5)	9.9–13.3	127 (11.0)	26 (14.4)	0.208
Unconjugated estriol	22 (1.7)	1.1–2.5	19 (1.7)	3 (1.7)	>0.999
AFP and inhibin A	50 (3.8)	2.9–4.9	43 (3.7)	7 (3.9)	0.835
Visual follow-up of the pregnancy	189 (14.2)	12.4–16.2	165 (14.3)	24 (13.3)	0.818
**BR. What are the main barriers to pregnant women receiving genetic care?**					
Shortage of specialists	567 (42.6)	40.0–45.3	506 (44.0)	61 (33.9)	0.012
Inadequate information for pregnant women	508 (38.2)	35.6–40.8	405 (35.2)	103 (57.2)	<0.001
Geographical distance	361 (27.1)	24.8–29.6	317 (27.6)	44 (24.4)	0.418
Financial constraints (services are fee-based only)	294 (22.1)	20.0–24.4	257 (22.3)	37 (20.6)	0.630
Insufficient awareness among health workers	160 (12.0)	10.4–13.9	120 (10.4)	40 (22.2)	<0.001
**RR. What are your reasons for not referring pregnant women for invasive prenatal diagnosis (e.g., amniocentesis)?**					
Risk of complications (pregnancy loss, infection, etc.)	709 (53.3)	50.6–56.0	604 (52.5)	105 (58.3)	0.149
Patients' fear and refusal of the procedure	634 (47.7)	45.0–50.4	547 (47.6)	87 (48.3)	0.873
Ethical or legal issues (difficulty obtaining permission)	181 (13.6)	11.9–15.6	154 (13.4)	27 (15.0)	0.559
Patient's socioeconomic situation (cannot afford it)	164 (12.3)	10.7–14.2	143 (12.4)	21 (11.7)	0.903
Lack of equipment or specialists	150 (11.3)	9.7–13.1	130 (11.3)	20 (11.1)	>0.999
Difficulty processing and interpreting results	146 (11.0)	9.4–12.8	124 (10.8)	22 (12.2)	0.608
Insufficient experience or knowledge of health workers	108 (8.1)	6.8–9.7	92 (8.0)	16 (8.9)	0.661
Other reasons	134 (10.1)	8.6–11.8	116 (10.1)	18 (10.0)	>0.999

Percentages sum to more than 100 because several answers were allowed. Wilson 95% CI. p, Fisher's exact test (primary care versus hospital or perinatal unit), unadjusted. Additional summary (analysis file, n = 1331): 97 respondents (7.3%) selected all three relevant orders and no distractor, they recognized a mean of 2.0 of five diagnostic methods; 79 (5.9%) named all five.

Table S2. Checklist for Reporting Results of Internet E-Surveys (CHERRIES)

Item category	Checklist item	Reported
Design	Describe survey design	Cross-sectional open online survey; convenience sample of clinical staff in antenatal care (Section 2.1–2.2).
IRB approval and informed consent	IRB approval	Local Bioethics Commission, Khoja Akhmet Yassawi University, protocol No. 4/26 (8 May 2026), conclusion No. 216 (29 May 2026) (Section 2.6; S2).
	Informed consent	Opening page stated the purpose, intended use of data and anonymity; electronic consent required to proceed (item C1).
	Data protection	No names, contact details, facility identifiers or IP-based identifiers collected; data held by the study team.
Development and pre-testing	Development and testing	Items mapped to national orders; reviewed for clarity by two obstetrician-gynecologists and one public health specialist (Section 2.3). No formal pilot.
Recruitment process	Open or closed survey	Open survey; link distributed through the regional health administration.
	Contact mode	Initial contact online through regional health administration channels.
	Advertising	No public advertising; invitation by regional health administration only.
Survey administration	Web/E-mail	Web form (Google Forms); data captured automatically.
	Context	Invitation distributed to facilities of all levels in Turkistan region.
	Mandatory/voluntary	Voluntary participation.
	Incentives	None.
	Time/date	1 June to 30 July 2026.
	Randomisation of items	No; fixed order in four blocks.
	Adaptive questioning	No.
	Number of items	38 items (35 content items, 3 respondent items).
	Number of screens	Four content blocks after the consent and respondent items.
	Completeness check	All items mandatory; a form could be submitted only when complete.
	Review step	Not specified in the form settings reported here.
Response rates	Unique site visitor	Not recorded by the platform; view rate cannot be calculated.
	View rate	Not available.
	Participation rate	Not available (views not recorded).
	Completion rate	1331 forms submitted; all complete (mandatory items).
Preventing multiple entries	Cookies used	No.
	IP check	No (IP addresses not collected).
	Log file analysis	Timestamps checked: all 1331 distinct.
	Registration	No registration; 13 repeated full response profiles (19 additional records) retained in the primary analysis and removed in a sensitivity analysis.
Analysis	Handling of incomplete questionnaires	Not applicable (no incomplete submissions).
	Questionnaires submitted with an atypical timestamp	Completion time not recorded; not assessed.
	Statistical correction	No weighting; sample described as a convenience sample; no regional workforce denominator was obtained, so coverage cannot be estimated.

Checklist from Eysenbach (J. Med. Internet Res. 2004, 6, e34).

Table S3. Knowledge items by level of care

Code	Item	Correct, n (%) N = 1331	95% CI	Primary care, % correct	Hospital or perinatal, % correct	p	Most frequent incorrect answer, n (%)
K1	What are the main aims of prenatal genetic screening?	1224 (92.0)	90.4–93.3	92.1	91.1	0.768	Determining fetal sex, 55 (4.1)
K2	Which stages of prenatal screening during pregnancy are the most important?	1195 (89.8)	88.0–91.3	89.8	89.4	0.224	Third trimester, 73 (5.5)
K3	What is karyotyping?	836 (62.8)	60.2–65.4	61.7	69.4	0.147	A method for detecting gene mutations, 243 (18.3)
K4	Which first-trimester maternal serum biochemical markers are used in Kazakhstan to detect developmental defects and chromosomal anomalies?	776 (58.3)	55.6–60.9	59.6	50.6	0.075	PAPP-A, β-hCG, AFP, 477 (35.9)
K5	Trisomy (Down syndrome) is associated with an anomaly of which chromosome?	772 (58.0)	55.3–60.6	56.6	67.2	0.022	X chromosome, 269 (20.2)
K6	Trisomy (Patau syndrome) is associated with an anomaly of which chromosome?	758 (56.9)	54.3–59.6	56.3	61.1	0.763	Chromosome 18, 205 (15.4)
K7	Trisomy (Edwards syndrome) is associated with an anomaly of which chromosome?	686 (51.5)	48.9–54.2	51.2	53.9	0.784	Chromosome 13, 247 (18.6)
K8	In which week of pregnancy is the first prenatal ultrasound screening performed?	652 (49.0)	46.3–51.7	50.6	38.3	<0.001	9^{+0}–12^{+6} weeks, 350 (26.3)
K10	In which week of pregnancy is the second prenatal ultrasound screening performed?	483 (36.3)	33.7–38.9	36.9	32.8	0.007	18^{+0}–21^{+6} weeks, 401 (30.2)
K11	Which examinations make up combined prenatal screening in the first trimester?	483 (36.3)	33.7–38.9	n/a	n/a	n/a	Ultrasound alone, 292 (21.9)
K12	In what unit are the results of first-trimester (combined) prenatal screening interpreted?	218 (16.4)	14.5–18.5	13.4	35.6	<0.001	IU/L, 499 (37.5)
K9	From what age are pregnant women considered at high risk of congenital and hereditary pathology?	148 (11.1)	9.5–12.9	9.8	19.4	0.003	From 35 years, 550 (41.4)

Proportion correct from the analysis file (n = 1331); K9 is shown against the national key (37 years). p, Fisher's exact test across all response options, primary care versus hospital or perinatal unit (export, n = 1330). K11 was scored all-or-nothing from a multiple-response list, so workplace percentages are not available from the export.

Table S4. Item responses by level of care

Primary care versus hospital or perinatal unit (institutional export; Fisher's exact test, unadjusted).

Response	Primary care, n (%) N = 1150	Hospital or perinatal, n (%) N = 180
**AW. Do you know whether a medical genetics service operates in the region where you work? (p = 0.059)**		
Yes, fully	756 (65.7)	134 (74.4)
Partly	237 (20.6)	30 (16.7)
Not aware	157 (13.7)	16 (8.9)
**K1. What are the main aims of prenatal genetic screening? (p = 0.768)**		
Assessing the risk of chromosomal anomalies	1059 (92.1)	164 (91.1)
Determining fetal sex	46 (4.0)	9 (5.0)
Assessing placental function	45 (3.9)	7 (3.9)
**K8. In which week of pregnancy is the first prenatal ultrasound screening performed? (p = <0.001)**		
9^{+0}–12^{+6} weeks	301 (26.2)	49 (27.2)
10^{+0}–14^{+6} weeks	118 (10.3)	19 (10.6)
11^{+0}–13^{+6} weeks	582 (50.6)	69 (38.3)
12^{+0}–14^{+6} weeks	149 (13.0)	43 (23.9)
**K10. In which week of pregnancy is the second prenatal ultrasound screening performed? (p = 0.007)**		
16^{+0}–20^{+0} weeks	183 (15.9)	32 (17.8)
18^{+0}–21^{+6} weeks	331 (28.8)	70 (38.9)
19^{+0}–21^{+0} weeks	424 (36.9)	59 (32.8)
19^{+6}–21^{+0} weeks	212 (18.4)	19 (10.6)
**K4. Which first-trimester maternal serum biochemical markers are used in Kazakhstan to detect developmental defects and chromosomal anomalies? (p = 0.075)**		
PAPP-A, β-hCG	685 (59.6)	91 (50.6)
PAPP-A, β-hCG, AFP	401 (34.9)	76 (42.2)
Inhibin A	20 (1.7)	6 (3.3)
Unconjugated estradiol	44 (3.8)	7 (3.9)
**K3. What is karyotyping? (p = 0.147)**		
A method for studying chromosome number and structure	710 (61.7)	125 (69.4)
A method for detecting gene mutations	213 (18.5)	30 (16.7)
Assessing fetal developmental stage by ultrasound	174 (15.1)	17 (9.4)
Assessing hormone levels in the body	53 (4.6)	8 (4.4)
**K9. From what age are pregnant women considered at high risk of congenital and hereditary pathology? (p = 0.003)**		
From 30 years	161 (14.0)	20 (11.1)
From 32 years	34 (3.0)	2 (1.1)
From 35 years	486 (42.3)	64 (35.6)
From 37 years	113 (9.8)	35 (19.4)
From 40 years	356 (31.0)	59 (32.8)
**K12. In what unit are the results of first-trimester (combined) prenatal screening interpreted? (p = <0.001)**		
MoM (multiple of the median)	154 (13.4)	64 (35.6)
IU/L	447 (38.9)	52 (28.9)
g/L	260 (22.6)	30 (16.7)
"MM" (abbreviation as printed on the form)	289 (25.1)	34 (18.9)
**A2. Do you think prenatal screening for fetal malformations and chromosomal anomalies should be mandatory for all pregnant women? (p = 0.103)**		
Yes, mandatory	970 (84.3)	156 (86.7)
Yes, recommended	101 (8.8)	8 (4.4)
Only when indicated	53 (4.6)	13 (7.2)
No	26 (2.3)	3 (1.7)

**Response**	**Primary care, n (%)**<br>**N = 1150**	**Hospital or perinatal, n (%)**<br>**N = 180**
**A1. What is your attitude to referring pregnant women at risk of congenital and hereditary pathology to a geneticist? (p = 0.127)**		
I consider it mandatory	1004 (87.3)	157 (87.2)
Recommended but not mandatory	97 (8.4)	9 (5.0)
Not needed, should be voluntary	27 (2.3)	11 (6.1)
Negative	22 (1.9)	3 (1.7)
**TOP. In your opinion, is termination of pregnancy on medical grounds appropriate when a severe untreatable hereditary disease is detected? (p = 0.141)**		
Yes	898 (78.1)	131 (72.8)
Difficult to answer	209 (18.2)	44 (24.4)
No	43 (3.7)	5 (2.8)
**PAW. How do you rate the population's awareness of what medical genetics services offer? (p = 0.532)**		
High	513 (44.6)	84 (46.7)
Moderate	554 (48.2)	80 (44.4)
Low	83 (7.2)	16 (8.9)
**AC. How do you rate the accessibility of medical genetic care for pregnant women in your region? (scale 1–5) (p = 0.013)**		
1 – Not accessible	132 (11.5)	17 (9.4)
2 – Accessible with difficulty	103 (9.0)	7 (3.9)
3 – Partly accessible	180 (15.7)	27 (15.0)
4 – Generally accessible	434 (37.7)	62 (34.4)
5 – Fully accessible	301 (26.2)	67 (37.2)
**A5. In your opinion, is invasive prenatal diagnosis necessary when indicated? (p = 0.901)**		
Necessary, mandatory	1061 (92.3)	169 (93.9)
With the pregnant woman's consent (free text)	3 (0.3)	0 (0.0)
Not necessary	80 (7.0)	11 (6.1)
I do not know (free text)	6 (0.5)	0 (0.0)
**A4. Do you think developing medical genetics services is important for the country? (p = 0.348)**		
Yes, very important	835 (72.6)	138 (76.7)
Yes, important	265 (23.0)	33 (18.3)
Difficult to answer	40 (3.5)	6 (3.3)
No, not important	10 (0.9)	3 (1.7)
**A6. How confident are you in your knowledge of prenatal genetic screening for fetal malformations and chromosomal anomalies? (p = 0.483)**		
Fully confident	441 (38.3)	76 (42.2)
I know the main aspects	218 (19.0)	33 (18.3)
I need further training	448 (39.0)	68 (37.8)
I do not understand the topic	43 (3.7)	3 (1.7)
**A3. Do you think introducing genetic counselling reduces the birth rate of children with hereditary diseases? (p = 0.641)**		
Yes	1012 (88.0)	157 (87.2)
Maybe (free text)	8 (0.7)	0 (0.0)
No	129 (11.2)	23 (12.8)
Did not understand (free text)	1 (0.1)	0 (0.0)
**P7. In the past year, have you referred patients to a medical geneticist for consultation? (p = <0.001)**		
Frequently	507 (44.1)	62 (34.4)
Sometimes	381 (33.1)	53 (29.4)
Did not refer	262 (22.8)	65 (36.1)
**P5. Do you use the results of medical genetic tests in your practice? (p = 0.004)**		
Yes, regularly	571 (49.7)	70 (38.9)
Sometimes	409 (35.6)	68 (37.8)
Never	170 (14.8)	42 (23.3)
**P4. Do you talk with patients about the importance of prenatal and neonatal screening? (p = 0.060)**		

Response	Primary care, n (%) N = 1150	Hospital or perinatal, n (%) N = 180
Frequently	643 (55.9)	93 (51.7)
Sometimes	401 (34.9)	59 (32.8)
Never	103 (9.0)	28 (15.6)
Of course (free text)	3 (0.3)	0 (0.0)
**P1. Do you recommend molecular genetic testing to patients when indicated? (p = 0.428)**		
Yes	847 (73.7)	138 (76.7)
Sometimes	218 (19.0)	27 (15.0)
No	85 (7.4)	15 (8.3)
**SRC. Where do you get information about prenatal screening during pregnancy?**		
Clinical guidelines	589 (51.2)	78 (43.3)
Scientific conferences or seminars	152 (13.2)	36 (20.0)
Colleagues	226 (19.7)	27 (15.0)
Internet or social media	179 (15.6)	33 (18.3)
"Always" (free text)	3 (0.3)	3 (1.7)
"Sometimes" (free text)	1 (0.1)	1 (0.6)
"Polyclinic" (free text)	0 (0.0)	1 (0.6)
"From the district nurse" (free text)	0 (0.0)	1 (0.6)
**P2. Do you talk with couples planning a pregnancy about the risk of hereditary diseases? (p = <0.001)**		
Yes, regularly	826 (71.8)	107 (59.4)
Sometimes	262 (22.8)	47 (26.1)
No	62 (5.4)	26 (14.4)
**P6. How often do you receive patients' molecular genetic test results (e.g., when a hereditary disease is suspected)? (p = 0.028)**		
Frequently	510 (44.3)	84 (46.7)
Rarely	494 (43.0)	62 (34.4)
Never	146 (12.7)	34 (18.9)
**P3. Do you advise patients to undergo NIPT (non-invasive prenatal testing) when indicated? (p = 0.330)**		
Yes, always	735 (63.9)	109 (60.6)
Sometimes	334 (29.0)	53 (29.4)
Never	81 (7.0)	18 (10.0)
**TR. Have you attended training or seminars on medical genetics in the last 3 years? (p = 0.013)**		
Yes	364 (31.7)	74 (41.1)
No	786 (68.3)	106 (58.9)
**K5. Trisomy (Down syndrome) is associated with an anomaly of which chromosome? (p = 0.022)**		
Chromosome 21	651 (56.6)	121 (67.2)
Chromosome 13	189 (16.4)	16 (8.9)
Chromosome 18	72 (6.3)	9 (5.0)
X chromosome	235 (20.4)	34 (18.9)
No answer	3 (0.3)	0 (0.0)
**K7. Trisomy (Edwards syndrome) is associated with an anomaly of which chromosome? (p = 0.784)**		
Chromosome 18	589 (51.2)	97 (53.9)
Chromosome 13	212 (18.4)	35 (19.4)
Chromosome 21	173 (15.0)	26 (14.4)
X chromosome	172 (15.0)	22 (12.2)
No answer	4 (0.3)	0 (0.0)
**K6. Trisomy (Patau syndrome) is associated with an anomaly of which chromosome? (p = 0.763)**		
Chromosome 13	648 (56.3)	110 (61.1)
Chromosome 18	180 (15.7)	25 (13.9)
Chromosome 21	166 (14.4)	24 (13.3)
X chromosome	152 (13.2)	21 (11.7)

Response	Primary care, n (%)<br>N = 1150	Hospital or perinatal, n (%)<br>N = 180
No answer	4 (0.3)	0 (0.0)
**K2. Which stages of prenatal screening during pregnancy are the most important? (p = 0.224)**		
First and second trimester	1033 (89.8)	161 (89.4)
Third trimester	66 (5.7)	7 (3.9)
Only before delivery	20 (1.7)	7 (3.9)
Screening is not needed during pregnancy	31 (2.7)	5 (2.8)

Multiple-response items are in Table S1. Comparisons are unadjusted and exploratory; with 30 tests, about 1.5 would be expected to reach $p < 0.05$ by chance.

Table S5. Former attitude composite

Measure	Attitude composite, 6 items (0–12)
Items	A1–A6 coded 2/1/0 (Supplementary Material S1)
Mean ± SD (% of maximum)	9.96 ± 1.96 (83.0)
Median (interquartile range)	10 (9–11)
Favourable (≥80% of maximum), n (%)	906 (68.1)
Cronbach's alpha	0.647
KMO; eigenvalues > 1	0.742; 1
aOR for recent training, favourable attitude (95% CI)	1.67 (1.26–2.21)

n = 1331. The composite is not used as an outcome, because its coding ranked "mandatory" above "recommended", it mixes confidence (A6) with attitudes, and it has a ceiling effect. It is retained only as a covariate in the practice models of the main text.

Table S6. Selected exploratory cross-tabulations

Column percentages; p as reported in the institutional cross-tabulation (n = 1330). These comparisons were not pre-specified and are not adjusted for multiple testing or confounding.

a. Self-rated confidence (A6) by genetics training in the previous 3 years (TR) (p <.001)

Confidence \ Training	No	Yes	Total
Knows the main aspects	175 (19.6%)	76 (17.4%)	251 (18.9%)
Does not understand the topic	38 (4.3%)	8 (1.8%)	46 (3.5%)
Fully confident	246 (27.6%)	271 (61.9%)	517 (38.9%)
Needs further training	433 (48.5%)	83 (18.9%)	516 (38.8%)
Total	892 (100.0%)	438 (100.0%)	1330 (100.0%)

b. Importance of developing genetics services (A4) by training (TR) (p = 0.001)

Importance \ Training	No	Yes	Total
Difficult to answer	33 (3.7%)	13 (3.0%)	46 (3.5%)
No, not important	11 (1.2%)	2 (0.5%)	13 (1.0%)
Yes, important	225 (25.2%)	73 (16.7%)	298 (22.4%)
Yes, very important	623 (69.8%)	350 (79.9%)	973 (73.2%)
Total	892 (100.0%)	438 (100.0%)	1330 (100.0%)

c. Awareness of a local genetics service (AW) by referral to a geneticist in the past year (P7) (p <.001)

Awareness \ Referral	Frequently	Did not refer	Sometimes	Total
Partly aware	68 (12.0%)	100 (30.6%)	99 (22.8%)	267 (20.1%)
Fully aware	447 (78.6%)	163 (49.8%)	280 (64.5%)	890 (66.9%)
Not aware	54 (9.5%)	64 (19.6%)	55 (12.7%)	173 (13.0%)
Total	569 (100.0%)	327 (100.0%)	434 (100.0%)	1330 (100.0%)

d. Awareness of a local genetics service (AW) by perceived accessibility, 1–5 (AC) (p <.001)

Awareness \ Accessibility	1	2	3	4	5	Total
Partly aware	23 (15.4%)	32 (29.1%)	78 (37.7%)	98 (19.8%)	36 (9.8%)	267 (20.1%)
Fully aware	93 (62.4%)	49 (44.5%)	93 (44.9%)	343 (69.2%)	312 (84.8%)	890 (66.9%)
Not aware	33 (22.1%)	29 (26.4%)	36 (17.4%)	55 (11.1%)	20 (5.4%)	173 (13.0%)
Total	149 (100.0%)	110 (100.0%)	207 (100.0%)	496 (100.0%)	368 (100.0%)	1330 (100.0%)

e. Unit chosen for interpreting combined-screening results (K12) by use of genetic test results (P5) (p = 0.036)

Unit \ Use of results	Never	Yes, regularly	Sometimes	Total
IU/L	69 (32.5%)	253 (39.5%)	177 (37.1%)	499 (37.5%)
"MM"	54 (25.5%)	170 (26.5%)	99 (20.8%)	323 (24.3%)
MoM	40 (18.9%)	99 (15.4%)	79 (16.6%)	218 (16.4%)
g/L	49 (23.1%)	119 (18.6%)	122 (25.6%)	290 (21.8%)
Total	212 (100.0%)	641 (100.0%)	477 (100.0%)	1330 (100.0%)

Among respondents trained in the previous three years, 61.9% described themselves as fully confident in their knowledge, compared with 27.6% of those not trained (Table S6a), although trained and untrained respondents did not differ in objective knowledge (main text, Table 6).

Figure S1. Location of Turkistan region relative to the national medical genetics services

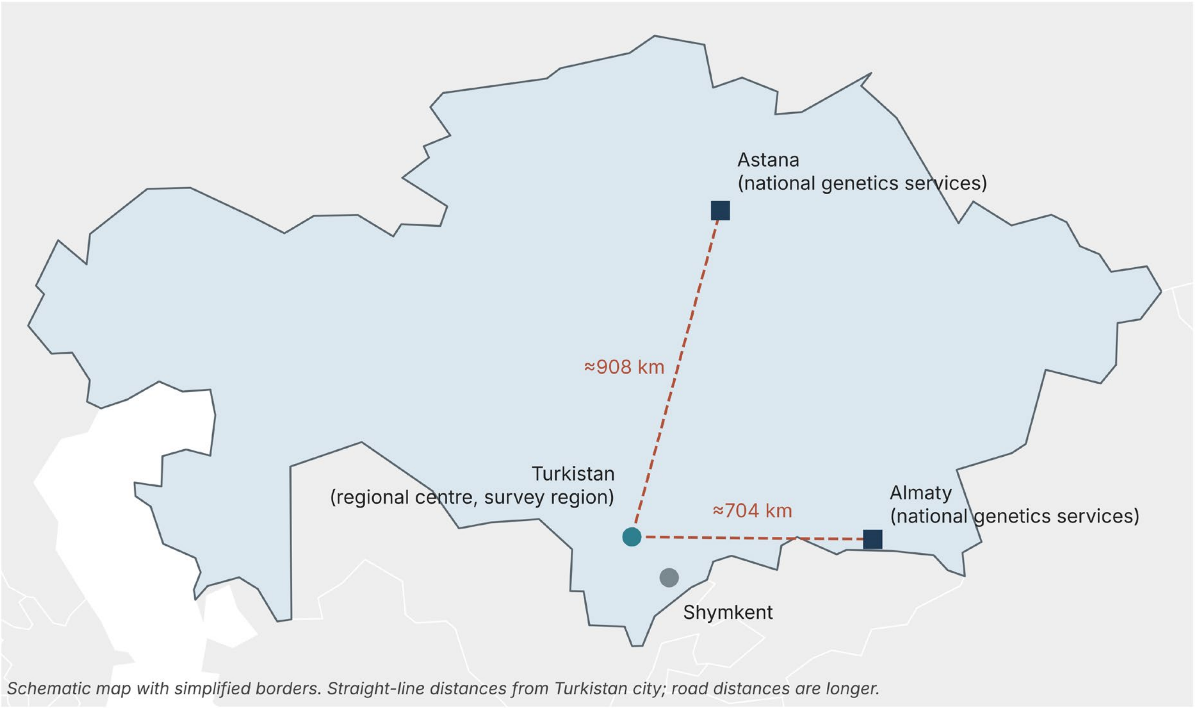

Schematic map with simplified borders. Dashed lines show straight-line distances from Turkistan city to Almaty (≈704 km) and Astana (≈908 km); road distances are longer.

Figure S2. Directed acyclic graph for the practice models

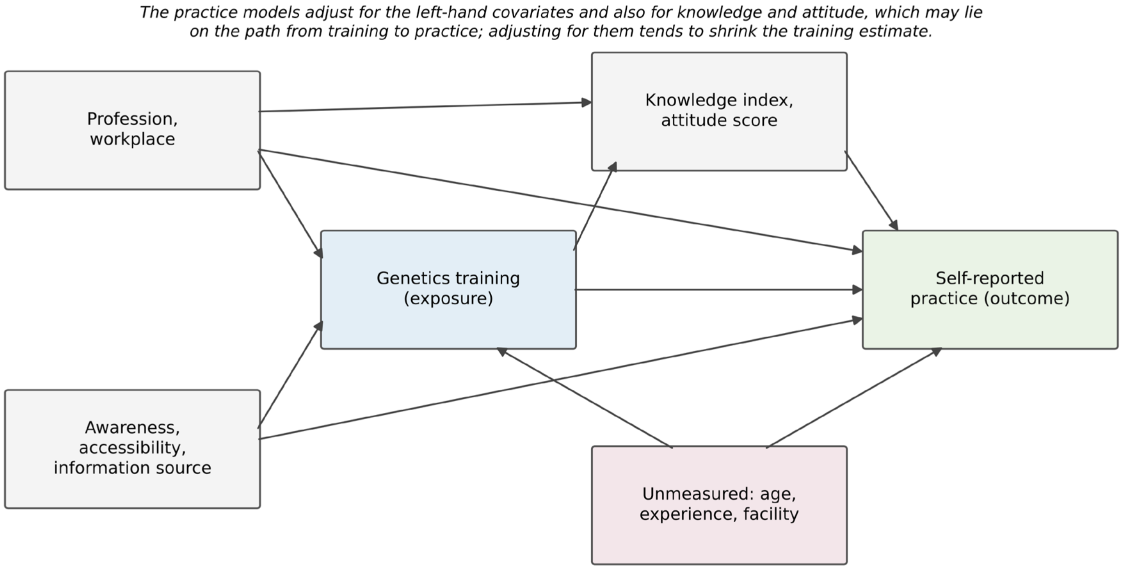

Training is the exposure and self-reported good practice the outcome. The practice models adjust for profession, workplace, awareness, accessibility and information source, and also for the knowledge index and the former attitude score. These two may lie on the path from training to practice, so adjusting for them tends to shrink the training estimate. Age, experience and facility were not recorded and remain unmeasured confounders.
