# Supplementary material for: Knowledge, Attitudes and Practices Regarding Prenatal Screening and Genetic Diagnosis of Chromosomal Anomalies Among Health Workers in Turkistan Region, Kazakhstan: A Cross-Sectional Survey of a Predominantly Primary-Care Workforce
Source: Healthcare (Basel). 2026 Nov 5;14(21):3776. doi: 10.3390/healthcare14213776 (PMC13762700; doi:10.3390/healthcare14213776)
Supplement: Supplementary file 1 [file healthcare-14-03776-s001.zip › Supplementary_Material_S2_Ethics_healthcare-4613524.pdf]

Supplementary Material S2

Healthcare manuscript ID healthcare-4613524. Ethics approval documents for the study.

Contents: (1) English translation of the conclusion of the Local Bioethics Commission of Khoja Akhmet Yassawi International Kazakh-Turkish University, No. 216 of 29 May 2026; (2) scan of the original conclusion in Kazakh; (3) scan of the KazREC certificate of the commission.

(1) English translation

Appendix 02-007/01.0

Khoja Akhmet Yassawi International Kazakh-Turkish University

Outgoing No. 216 Date: 29.05.2026

CONCLUSION OF THE LOCAL COMMISSION ON BIOETHICS

1	Full name of the doctoral student	Torgauytova Zhansaya Ermuratovna
2	Doctoral specialty (educational programme)	8D10111 – Public Health
3	Period of doctoral study	2025–2028 academic years
4	Dissertation topic and date of approval	Improving the organization of medical genetic care for pregnant women in a region with a high birth rate (the case of Turkistan region). Order No. TO 223 of 25.12.2025
5	Scientific supervisors: full name, place of work and position, academic degree, citizenship	Scientific supervisor: PhD, Associate Professor Ayazbekov Ardak Kerimkhanovich. Foreign scientific supervisor: Professor, Dr. Seçil Özkan
6	Objects of research	Staff of medical organizations of all levels in Turkistan region (perinatal centres, primary health care organizations, district hospitals, feldsher-midwife posts) (for the KAP questionnaire); medical data of pregnant women (ultrasound examination, biochemical screening, invasive prenatal diagnosis)
7	Violations in planning, evaluating, selecting and conducting the research	No violations identified
8	Violations in disseminating the research results	No violations identified
9	How were the rights, safety and health of research objects protected (for new natural and environmental objects)?	In accordance with the legislation of the Republic of Kazakhstan and international ethical standards
10	Date and protocol number of the positive decision of the Local Commission on Bioethics	08.05.2026, protocol No. 4/26
11	Validity of the decision of the Local Commission on Bioethics	08.05.2027

Chair of the Local Commission on Bioethics, Khoja Akhmet Yassawi International Kazakh-Turkish University, Candidate of Medical Sciences, Associate Professor [signature, seal] Oshibayeva A.E.

Prepared by: Asan A.A.

Note on chronology: the dissertation topic was approved on 25 December 2025, the Commission approved the study on 8 May 2026, and the survey was conducted in June–July 2026, after approval.

Translated from Kazakh by the authors.

(2) Original conclusion (Kazakh)

Қосымша 02-007/01.0

Қожа Ахмет Ясауи атындағы Халықаралық қазақ - түрік университеті

Международный казахско - турецкий университет имени Ходжи Ахмеда Ясави

Шығыс №: 216
Күні: 29.05.2026

Биоэтика жөніндегі жергілікті комиссиясының
ҚОРЫТЫНДЫСЫ

1	Докторанттың Т.А.Ә.	Торгауытова Жансая Ермуратовна
2	Докторантура мамандығы (білім беру бағдарламасы)	8D10111 – Қоғамдық денсаулық сақтау
3	Докторантурада оқу кезеңі	2025–2028 оқу жылдары
4	Диссертация тақырыбы, бекіту мерзімі	Туу деңгейі жоғары өңірде жүкті әйелдерге медико-генетикалық көмекті ұйымдастыруды жетілдіру (Түркістан облысының мысалында)<br>Бұйрық №ТО 223, күні: 25.12.2025 ж.
5	Ғылыми кеңесшілер туралы деректер – Т.А.Ә. (болған жағдайда), жұмыс орны және лауазымы, ғылыми дәрежелері, азаматтығы	Ғылыми жетекші: PhD, қауымдастырылған профессор Аязбеков Ардак Керимханович<br>Шетелдік ғылыми жетекші: профессор, доктор Сечил Өзкан
6	Зерттеу объектілері	Түркістан облысының әртүрлі деңгейдегі медицина мекемелерінің (перинаталды орталықтар, алғашқы медициналық-санитарлық ұйымдар, аудандық ауруханалар, ФАП) қызметкерлері (КАР-сауалнама үшін); Жүкті әйелдердің медициналық деректері (ультрадыбыстық зерттеу, биохимиялық скрининг, ИПД);
7	Ғылыми зерттеулерді жоспарлау, бағалау, іріктеу және жүргізу процесіндегі бұзушылықтар	Бұзушылықтар анықталмады
8	Ғылыми зерттеу нәтижелерін тарату процесіндегі бұзушылықтар	Бұзушылықтар анықталмады
9	Зерттеу объектілерінің (жаңа табиғат пен тіршілік ортасының объектілері болғанда) құқықтарын, қауіпсіздігі мен саулығын қорғау қалай жүргізілді?	Қазақстан Республикасының заңнамасына және халықаралық этикалық стандарттарға сәйкес
10	Биоэтика жөніндегі жергілікті комиссиясының оң шешімі берілген уақыт және хаттама №	08.05.2026 жыл, хаттама №4/26
11	Биоэтика жөніндегі жергілікті комиссиясының шешімі жарамды уақыт	08.05.2027 жыл

Қ.А. Ясауи атындағы ХҚТУ, Биоэтика жөніндегі жергілікті комиссиясының Төрайымы, м.ғ.к., қауымд.профессор **Ошибаева А.Е.**

Орындаған: Асан А.А.

(3) **KazREC certificate of the Local Commission on Bioethics (registration No. XXV-R, full certification, 05.12.2025–05.12.2028)**

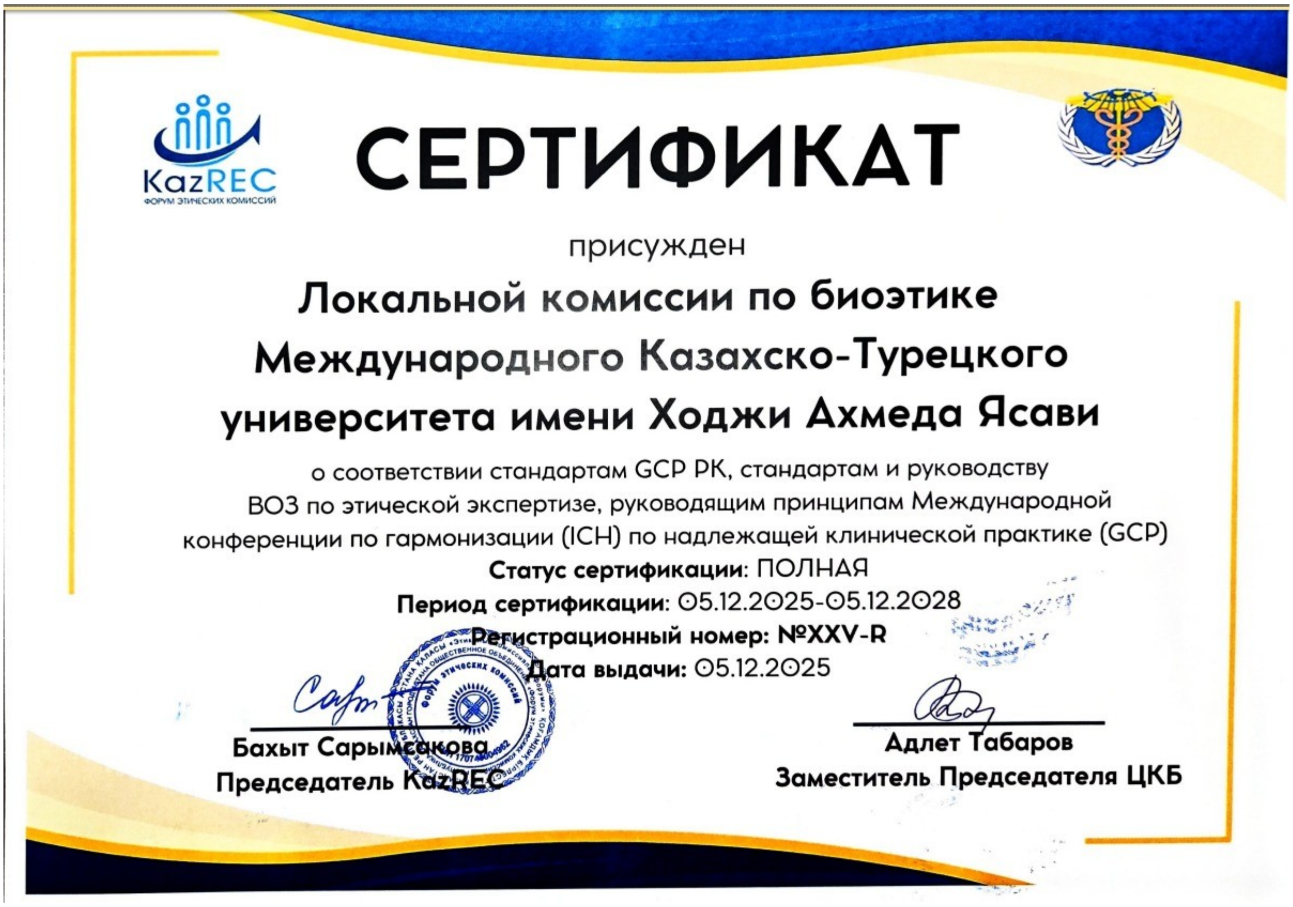

KazREC
ФОРУМ ЭТИЧЕСКИХ КОМИССИЙ

СЕРТИФИКАТ

присужден

Локальной комиссии по биоэтике Международного Казахско-Турецкого университета имени Ходжи Ахмеда Ясави

о соответствии стандартам GCP РК, стандартам и руководству ВОЗ по этической экспертизе, руководящим принципам Международной конференции по гармонизации (ICH) по надлежащей клинической практике (GCP)

Статус сертификации: ПОЛНАЯ

Период сертификации: 05.12.2025-05.12.2028

Регистрационный номер: №XXV-R

Дата выдачи: 05.12.2025

Бахыт Сарымсакова
Председатель KazREC

Адлет Табаров
Заместитель Председателя ЦКБ
